# Characterization of the Reversible Intersystem Crossing Dynamics of Organic Photocatalysts Using Transient Absorption Spectroscopy and Time-Resolved Fluorescence Spectroscopy

**DOI:** 10.1021/acs.jpca.3c04780

**Published:** 2023-12-14

**Authors:** William Whitaker, Igor V. Sazanovich, Yonghwan Kwon, Woojin Jeon, Min Sang Kwon, Andrew J. Orr-Ewing

**Affiliations:** † School of Chemistry, University of Bristol, Cantock’s Close, Bristol BS8 1TS, U.K.; ‡ Department of Materials Science and Engineering, Seoul National University, Seoul 08826, Republic of Korea; § Central Laser Facility, Research Complex at Harwell, Science and Technology Facilities Council, Rutherford Appleton Laboratory, Harwell Oxford, Didcot, Oxfordshire OX11 0QX, U.K.

## Abstract

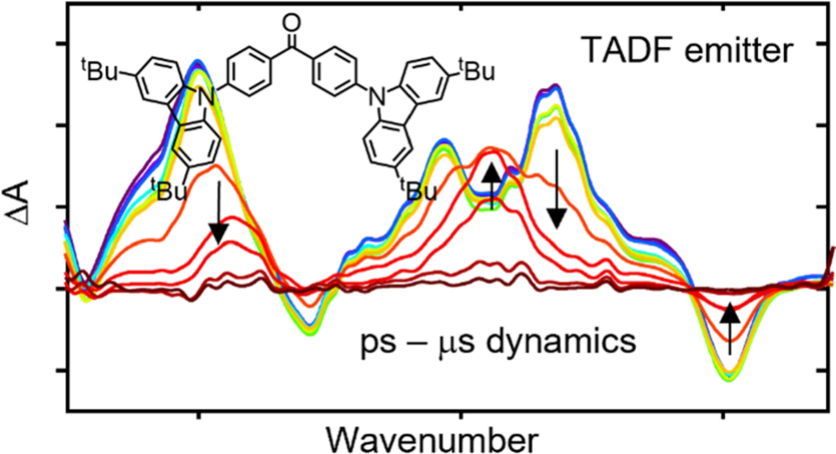

Thermally activated delayed fluorescence (TADF) emitters are molecules of interest as homogeneous organic photocatalysts (OPCs) for photoredox chemistry. Here, three classes of OPC candidates are studied in dichloromethane (DCM) or N,N-dimethylformamide (DMF) solutions, using transient absorption spectroscopy and time-resolved fluorescence spectroscopy. These OPCs are benzophenones with either carbazole (2Cz-BP and 2tCz-BP) or phenoxazine/phenothiazine (2PXZ-BP and 2PTZ-BP) appended groups and the dicyanobenzene derivative 4DP-IPN. Dual lifetimes of the S_1_ state populations are observed, consistent with reverse intersystem crossing (RISC) and TADF emission. Example fluorescence lifetimes in DCM are (5.18 ± 0.01) ns and (6.22 ± 1.27) μs for 2Cz-BP, (1.38 ± 0.01) ns and (0.32 ± 0.01) μs for 2PXZ-BP, and (2.97 ± 0.01) ns and (62.0 ± 5.8) μs for 4DP-IPN. From ground state bleach recoveries and time-correlated single photon counting measurements, triplet quantum yields in DCM are estimated to be 0.62 ± 0.16, 0.04 ± 0.01, and 0.83 ± 0.02 for 2Cz-BP, 2PXZ-BP, and 4DP-IPN, respectively. 4DP-IPN displays similar photophysical behavior to the previously studied OPC 4Cz-IPN. Independent of the choice of solvent, 4DP-IPN, 2Cz-BP, and 2tCz-BP are shown to be TADF emitters, whereas emission by 2PXZ-BP and 2PTZ-BP depends on the molecular environment, with TADF emission enhanced in aggregates compared to monomers. Behavior of this type is representative of aggregation-induced emission luminogens (AIEgens).

## 1 Introduction

Photoredox catalysis is increasingly recognized as a valuable tool for chemical and materials synthesis because it can promote synthetically important transformations using low-cost and efficient light sources, while offering a lower toxicity alternative to some traditional synthetic reagents.^1−3^ The photocatalysts (PCs) used in photoredox strategies are electronically excited by the absorption of UV or visible light, and the resulting excited-state species typically have redox potentials of greater magnitude than the ground electronic state. The photoexcitation therefore facilitates electron transfer reactions that are otherwise thermodynamically unfavorable.^4,5^

Inorganic photoredox catalysts such as [Ru(bpy)_3_]^2+^ and *fac-*Ir(ppy)_3_ have been widely applied and extensively studied, and the mechanisms of their single-electron transfer (SET) photoredox behavior are now well understood.^6−9^ Continuing efforts seek to refine the design and performance of metal-based PCs, including the in situ generation of Cu(I) halides via reduction of Cu(II) complexes for click chemistry^10,11^ and use of *fac-*Ir(ppy)_3_ for better control of living radical polymerization.^12^ Despite the popularity of these metal-based PCs, there are several incentives to replace them with organic photocatalysts (OPCs).^13−15^ Motivations include the natural scarcity of the precious metals that are critical to the function of the well-established PCs, toxicity (which may also influence biocompatibility in certain applications), and contamination of synthetic products.^16^ Replacement of metal-based PCs by OPCs also offers wider benefits beyond more sustainable synthesis. For example, an organo-photoredox-catalyzed free-radical pathway for hydroformylation of aryl olefins reported by Wang and co-workers^17^ improved upon the established metal-catalyzed process, with greater regio- and chemoselectivity of product aldehydes, and eliminated the need for high pressures of toxic syngas. Chemoselective dehalogenation and carbon–carbon cross-coupling reactions using OPCs under mild conditions,^18^ as well as photoredox-catalyzed pressure-sensitive adhesive production initiated by visible light,^19−21^ further illustrate the scope for applications of OPCs in modern chemistry.

Organic photoredox catalysis exploits the enhanced redox behavior of OPCs in excited electronic states. Examples of excited-state oxidants are more common than excited-state reductants,^22,23^ with synthetically useful examples including erythrosine B for radical ring expansion,^24^ eosin-Y for radical cyclization,^25^ and carbazolyl derivatives in cross-coupling reactions.^26^ Catalysts with sufficiently reducing excited states to activate alkyl halides in oxidative quenching cycles (approximately −0.6 to −0.8 V vs SCE)^5^ are rarer but are desirable to avoid the use of sacrificial electron donors.^23,27^ Approaches to designing functional, strongly reducing OPCs include molecules based on N-aryl phenoxazine and phenothiazine derivatives,^27^ perylene,^22^ and N,N-diaryl dihydrophenazines.^28^ Characterization of these photoredox catalysts using transient absorption spectroscopy in our laboratory has highlighted the utility of photophysical studies to further inform and optimize the rational design of OPCs.^4,29−34^

**Figure 1:**
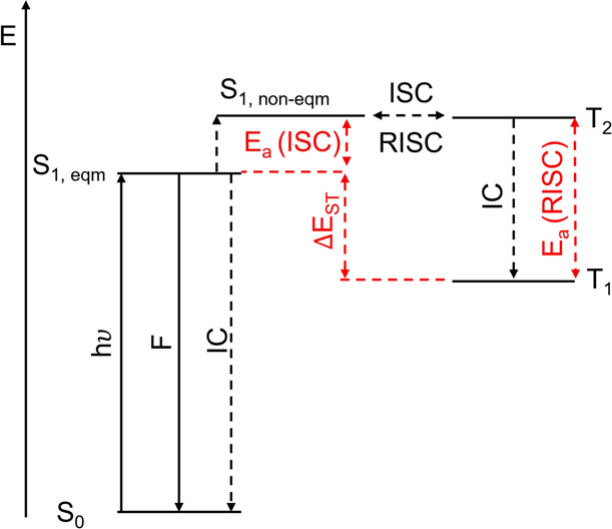
Energy level diagram for a TADF emitter. Solid and dashed black lines represent radiative and nonradiative processes, respectively. The singlet–triplet energy gap (Δ*E*_ST_) and activation energies of intersystem crossing (ISC) and reverse intersystem crossing (RISC) are represented by dashed red lines. Fluorescence (*F*) comprises both prompt and delayed components, as described in the main text. The illustrated mechanism considers ISC via an intermediate triplet state, instead of directly to the T_1_ state, and the need for distortion of the equilibrium S_1_ geometry to alter its electronic character to promote effective spin–orbit coupling.^43^

An emerging strategy for OPC design is to use compounds exhibiting thermally activated delayed fluorescence (TADF), which have long-lived excited states and redox potentials comparable to metal-centered photoredox catalysts.^35^ Originally described as *E*-type fluorescence,^36^ TADF has been known for over 50 years, and has been applied in fullerenes for oxygen sensing^37,38^ and more extensively in the development of high quantum efficiency, metal-free OLED displays for effective harvesting of triplet excitons.^39−42^ Typical photophysical behavior of a TADF emitter is shown in Figure 1.

Following photon absorption to populate an excited electronic state (typically S_1_), radiative and nonradiative relaxation channels repopulate the ground state on nanosecond time scales. Fluorescence emission on this time scale is termed prompt fluorescence (PF). Competitive intersystem crossing (ISC) into the triplet manifold is also observed, with growth of population in the T_1_ state facilitated by initial coupling with a higher energy intermediate triplet excited state.^43^ If the energy gap between the S_1_ and T_1_ states of the catalyst (Δ*E*_ST_) is sufficiently small that reverse intersystem crossing (RISC) is feasible at ambient temperature, the initial excited singlet state can be repopulated and subsequently relaxes to the ground state.^44,45^ The radiative component of this relaxation is termed delayed fluorescence (DF) and can be temporally resolved from the faster PF emission.

The efficient ISC and RISC dynamics of molecules showing TADF behavior encourages exploration of this class of compounds as potential OPC candidates, particularly in photoinduced electron transfer reactions.^35^ Structures designed around sulfone or carbazole moieties have been shown to catalyze free-radical polymerization of methacrylates, with their catalytic performance linked to their TADF behavior.^46^ Of various OPC architectures studied to date, derivatives of the known TADF emitter 2,4,5,6-tetra(carbazol-9-yl)benzene-1,3-dicarbonitrile (4Cz-IPN) have emerged as popular and versatile photocatalysts in synthetic chemistry.^47−51^

Studies of organic luminophores for OLED applications have revealed that TADF emission can be optimized by decreasing Δ*E*_ST_ to reduce the barrier for thermal activation of RISC and by ensuring that the rate constant for fluorescence (*k*_f_) is competitive with other photophysical processes.^52,53^ An effective way to reduce Δ*E*_ST_ is to minimize the overlap integral for the wave functions of the frontier orbitals by spatially separating the HOMO and the LUMO.^41,53,54^ This approach was successfully used by Adachi and co-workers, who designed a TADF molecule exhibiting high electroluminescence based on a phenoxazine-triphenyltriazine derivative.^55^ Subsequently, they also demonstrated efficient TADF from benzophenone derivatives termed “*luminous butterflies*”.^42^ These examples highlight that designing organic molecules with a twisted molecular architecture is an effective way to spatially separate the frontier molecular orbitals via disruption of a π-conjugated system, thereby reducing Δ*E*_ST_ and promoting TADF. To exploit twisted geometries, OPC designs with the HOMO localized on an electron donating unit (D) and the LUMO on an electron accepting unit (A), are becoming increasingly popular, giving rise to D–A molecular frameworks or D–A–D frameworks in the case of luminous butterflies.

A key advantage of an OPC with a D–A architecture is that the redox potential of the molecule depends on the relative energies of the donor and acceptor units. Hence, the ability to modify each of these motifs independently to tune the redox behavior increases the versatility of this type of scaffold and has inspired rational design of OPCs based on twisted D–A systems using moieties other than benzophenone.^56^ Moreover, by localizing the frontier orbitals over discrete donor and acceptor regions of a molecule, HOMO to LUMO electronic excitation acquires intramolecular charge transfer (ICT) character. The performance of the OPC can then be further manipulated through changes in its excited-state energetics and dynamics in solvents of different polarities.

**Figure 2:**
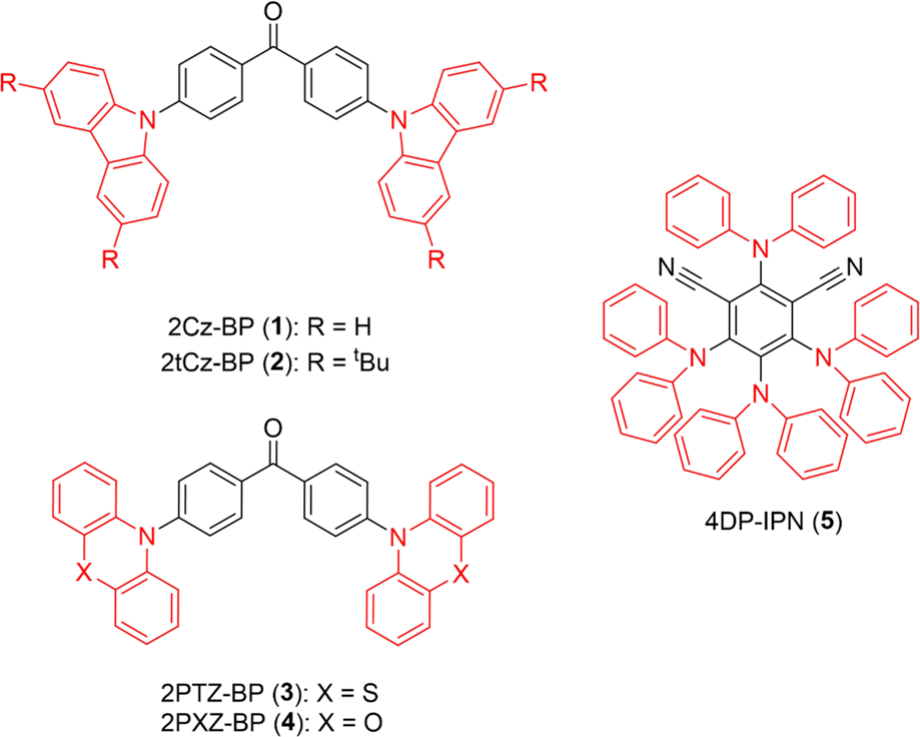
Structures of the OPCs examined here. (**1,2**) Carbazole-type D–A–D molecules, (**3,4**) phenothiazine/phenoxazine-type D–A–D molecules, and (**5**) dicyanobenzene-type D–A molecules. Donor motifs are colored red, and acceptor motifs are colored black.

Here, we explore the photophysics of five OPCs exhibiting donor–acceptor motifs by using transient absorption spectroscopy, supplemented by time-correlated single photon counting (TCSPC). The structures of these OPCs are shown in Figure 2. Four benzophenone-based catalysts (**1**–**4**) were selected, as they are representative of luminous butterflies. In addition, 4DP-IPN (**5**) was chosen because of its resemblance to 4Cz-IPN. Previous studies by Kwon and co-workers demonstrate that each of these OPCs can be used to catalyze atom transfer radical polymerization (ATRP) reactions via an oxidative quenching cycle.^57^ However, investigations of the underlying photodynamics in these species have not been reported. By understanding the excited-state dynamics, we aim to unravel the ISC/RISC behavior of these CT donor–acceptor types of OPCs in solution and evaluate typical excited-state lifetimes. Using this information, we can relate fundamental molecular properties to the observed excited-state behavior, and hence inform the design of functional OPCs based on TADF emitters for applications in chemical and materials synthesis.

## 2 Methods

Solutions of 2Cz-BP (1.0 mM), 2tCz-BP (1.25 mM), 2PTZ-BP (1.5 mM), 2PXZ-BP (2.5 mM), and 4DP-IPN (2.5 mM) were prepared in dichloromethane (DCM) and *N,N*-dimethylformamide (DMF) for transient electronic absorption spectroscopy (TEAS) and transient vibrational absorption spectroscopy (TVAS) experiments. The TEAS measurements spanned time intervals after UV excitation from 100 fs to 1.3 ns, whereas TVAS measurements extended to several microseconds.^58,59^ For TEAS measurements, an ultrafast 360 nm UV pump pulse was used to excite each solution, which was then probed using a broadband white-light continuum (WLC) pulse. For TVAS measurements, either 360 nm (2Cz-BP, 2tCz-BP and 2PTZ-BP) or 425 nm (2PXZ-BP and 4DP-IPN) ultrafast pump pulses were selected to excite the samples, before probing using a pair of synchronized broadband IR pulses together spanning the 1400–1800 cm^–1^ range. For TCSPC experiments, OPC solutions (10 μM, DCM and DMF) were excited at 377 nm, with fluorescence emission observed at 530 nm (2Cz-BP, 2tCz-BP and 4DP-IPN) and at 404 nm (2PTZ-BP and 2PXZ-BP). Complete descriptions of the experimental methods can be found in the Supporting Information (S1).

Interpretation of the experimental measurements was assisted by density functional theory (DFT) and time-dependent density functional theory (TDDFT) calculations at the ωB97XD/6-31+G(d) level of theory using the Tamm-Dancoff approximation (TDA), implemented in Gaussian 09.^60^ DFT was chosen for the electronic structure calculations on account of the large sizes of the studied OPCs. The long-range corrected functional ωB97XD was selected because it previously performed well in excited-state studies of comparable organic molecules exhibiting ICT character and TADF emission.^43^ Calculations were performed on isolated molecules. Visualization of calculated molecular structures and orbitals used the Avogadro molecular builder and visualization software.^61^

## 3 Results and Discussion

To explore the photodynamic behavior of compounds 1–5 and their propensities for RISC and TADF emission, several complementary spectroscopic techniques were used. Steady-state UV–visible absorption spectroscopy identified electronic transitions from the ground state (Section 3.1), the orbital characters of which were elucidated using TDDFT calculations. TEAS and TVAS measurements characterized which species and electronic states contributed to the excited-state dynamics (Section 3.3) and determined the time scales for nonadiabatic processes and electronic relaxation. Kinetics determined via transient absorption spectroscopy were supplemented by TCSPC measurements of S_1_-state radiative lifetimes for the OPCs (Section 3.2). Together, these time-resolved spectroscopy measurements revealed the influence of the solvent environment, which was further explored by using steady-state fluorescence spectroscopy (Section 3.4).

### 3.1 Excited-State Characterization

**Figure 3:**
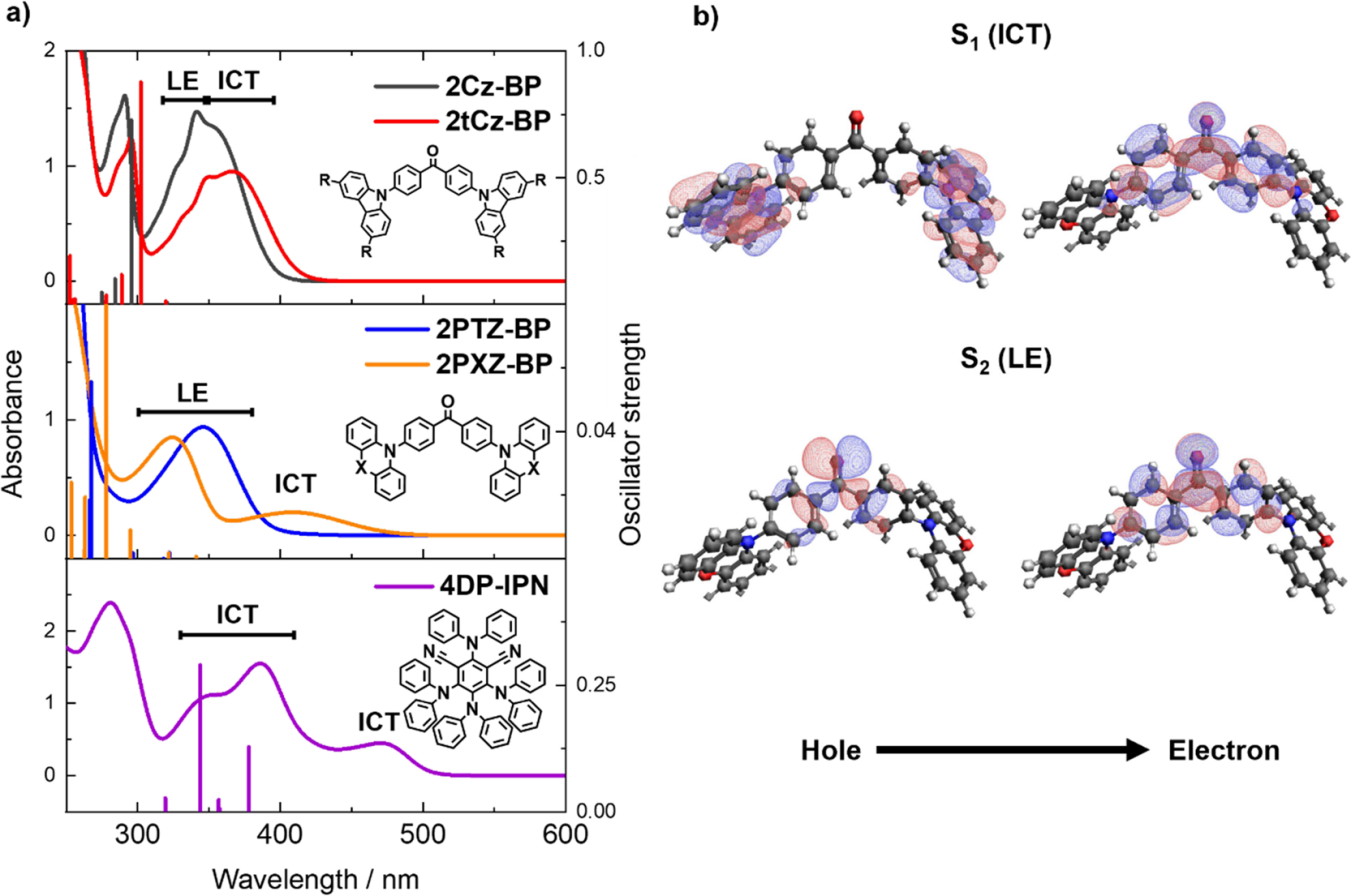
(a) UV–visible absorption spectra (solid lines) for 2.5 mM solutions of each photocatalyst overlaid with VEEs (bars) calculated at the ωB97XD/6-31+G(d) level of theory using the Tamm-Dancoff approximation. Spectra of carbazole-type and phenothiazine/phenoxazine-type D–A–D OPCs were recorded in DCM and the spectrum of 4DP-IPN was recorded in MeCN. (b) NTO analysis for 2PXZ-BP calculated at the ωB97XD/6-31+G(d) level of theory. The panel shows hole (left) and electron (right) orbital densities for the S_0_ to S_1_ electronic transition (top) and the S_0_ to S_2_ electronic transition (bottom).

Steady-state UV–visible absorption spectra of the five OPCs are shown in Figure 3 for solutions in either DCM or acetonitrile (MeCN). The experimental measurements are compared with TDDFT predictions of the wavelengths for the lowest vertical excitation energies (VEEs) of each molecule.

The steady-state absorption spectra in Figure 3 informed our selection of pump excitation wavelengths of 360 and 425 nm for transient absorption spectroscopy studies so that low-lying singlet excited states were accessed. UV–visible absorption spectra for the carbazole-type compounds reveal two absorption bands that are mostly resolved in the near-UV. The lower-energy, broad band centered around 375 nm is composed of two electronic transitions within the singlet-state manifold. TDDFT calculations, coupled with natural transition orbital (NTO) analysis, identify that the short-wavelength edge of the absorption band corresponds to excitation to an S_2_ state of locally excited (LE) electronic character, and we assign the longer-wavelength edge of the absorption band to an S_0_ to S_1_ ICT transition from the carbazole donor units to the benzophenone acceptor moiety. This S_1_ ICT character is confirmed by solvatochromic shifts in the steady-state fluorescence emission spectra of 2Cz-BP and 2tCz-BP recorded in solvents of different polarities (see Section S2.3 and Figure S7 of the Supporting Information). DFT methods typically fail to calculate accurately the energies and oscillator strengths of CT transitions,^62−64^ and this is true of the VEE values calculated in this study. For this reason, there is no corresponding S_0_ to S_1_ VEE explicitly shown in Figure 3 for 2Cz-BP and 2tCz-BP. However, it is reasonable to assume that a 360 nm pump wavelength will preferentially excite the long-wavelength edge of the absorption band, populating the S_1_ ICT state with excess vibrational energy.

The luminous butterflies, 2PTZ-BP and 2PXZ-BP, both exhibit absorption bands in the near-UV, centered around 345 and 325 nm, respectively, that correspond to LE transitions.^65^ However, at wavelengths exceeding 400 nm, the spectra differ, with 2PXZ-BP showing an additional absorption band that is not evident in the 2PTZ-BP spectrum. Computational analysis proposes this to be an ICT transition arising from donation of electron density from the phenoxazine moieties toward the benzophenone acceptor unit, with the phenoxazine groups in 2PXZ-BP being superior electron donors compared to phenothiazine in 2PTZ-BP.^66^ NTO analysis of 2PXZ-BP is shown in Figure 3b for the two lowest energy transitions. Significantly, there is no evidence of an ICT absorption band at wavelengths exceeding 400 nm for 2PTZ-BP in either the experimental spectrum or the DFT calculations. Hence, excitation using a 360 nm laser pulse facilitates a LE transition to the S_2_ state, with subsequent IC resulting in the population of the S_1_ ICT state that is not directly accessible optically.

The UV–visible absorption spectrum of 4DP-IPN differs from those of the four benzophenone-based catalysts, exhibiting three significant absorption bands across visible and near-UV wavelengths. Similar to the benzophenone-based compounds, the lowest energy absorption band is assigned to a donor–acceptor ICT transition, with excitation using a 425 nm pump pulse promoting an S_0_ to S_2_ transition. Following excitation to the S_2_ state, IC populates the S_1_ state from which fluorescence emission occurs. Figure S7 of Supporting Information shows solvent-dependent fluorescence emission spectra for 4DP-IPN.

### 3.2 Time-Resolved Fluorescence Spectroscopy

To characterize S_1_-state lifetimes of the OPCs, TCSPC experiments were performed for solutions in DCM and DMF. Prompt (τ_PF_) and delayed (τ_DF_) fluorescence time constants for each of the catalysts are presented in Table 1, with representative data traces and fits available in the Supporting Information (Section S4). To verify that the two components of fluorescence derive from the same species in solution, TCSPC measurements were made at selected detection wavelengths across the emission bands for two of the OPCs (2PTZ-BP and 2PXZ-BP). Further verification comes from the transient absorption spectroscopy measurements reported below.

**Table 1:** Fluorescence Lifetimes for the Five Studied Organic Photocatalysts in Dichloromethane and *N,N-*Dimethyl Formamide Obtained Using TCSPC

compound	time constant/ns			
	DCM		DMF	
	aτ_PF_	bτ_DF_	aτ_PF_	bτ_DF_
2Cz-BP	5.18 ± 0.01	6220 ± 1270	7.51 ± 0.01	3260 ± 360
2tCz-BP	8.66 ± 0.01	12600 ± 3600	7.80 ± 0.01	7900 ± 1300
2PTZ-BP	cIRF and 1.18 ± 0.02	350 ± 10	cIRF and 0.88 ± 0.04	310 ± 10
2PXZ-BP	cIRF and 1.38 ± 0.01	320 ± 10	cIRF and 1.07 ± 0.04	300 ± 20
4DP-IPN	2.97 ± 0.01	62000 ± 5800	3.43 ± 0.01	75700 ± 8600

a Unsparged solutions.

b Solutions sparged with argon for 10 min.

c Time constants smaller than the instrument response function (IRF) are reported as IRF limited.

The observation of fluorescence emission for each of the five compounds confirms that radiative decay pathways are competitive in each system, which is an important prerequisite for TADF behavior. Evidence of multiple temporally distinct fluorescence components for the OPCs is also representative of typical TADF emitter dynamics. Analysis of the emission decay traces for 4DP-IPN in DMF yields a prompt fluorescence lifetime of (3.43 ± 0.01) ns as well as a long-lived delayed fluorescence component of (75.7 ± 8.6) μs. These data indicate that the excited-state population resides in the S_1_ state for a few nanoseconds, before being distributed radiatively to the ground state and nonradiatively into the triplet manifold via efficient ISC, resulting in an S_1_ lifetime of 3.4 ns. From the T_1_ state, RISC then occurs to repopulate the S_1_ state, giving rise to the delayed component of fluorescence over long times.

For both carbazole-type catalysts, the TCSPC decay traces for the prompt and delayed components are separately well-fitted to functions with a single decay component. For 2Cz-BP in DMF, τ_PF_ = (7.51 ± 0.01) ns and τ_DF_ = (3.26 ± 0.36) μs, and 2tCz-BP exhibits fluorescence lifetimes of τ_PF_ = (7.80 ± 0.01) ns and τ_DF_ = (7.9 ± 1.3) μs in DMF.

For the luminous butterflies, 2PTZ-BP and 2PXZ-BP, the prompt TCSPC decay traces are fitted to functions with two decay components, rather than one as for other OPCs. For measurements in DMF, the two time constants for prompt fluorescence decay from 2PTZ-BP are <250 ps (IRF limited) and (0.88 ± 0.04) ns, whereas for 2PXZ-BP, the values are <250 ps (IRF limited) and (1.07 ± 0.04) ns. In the fits, the sub-IRF components have a greater weight than the approximately one nanosecond decay components most likely because of enhancement of the IRF signal by a Raman band of greater intensity than the weak fluorescence emission originating from the OPCs in this wavelength region. Comparison of these time constants with values obtained via TVAS for analogous photophysical processes (section 3.3) indicates the presence of two molecular environments in solution, an aggregated state and a free molecular state, as discussed further in section 3.4. Weak prompt fluorescence from solvated monomers occurs within the IRF time scale, but it is obscured by the aforementioned Raman scattering. The approximately 1 ns component is not observed using TVAS (Section 3.3 and Table 2), indicating an enhancement in fluorescence decay measurements. Both species have a delayed fluorescence time constant of approximately 300 ns in DMF, which is orders of magnitude smaller than the delayed fluorescence lifetimes shown by other OPC classes studied here but is still representative of TADF emission.

**Table 2:** Excited-State Lifetimes for the Five Studied Organic Photocatalysts in Dichloromethane and *N,N-D*imethyl Formamide Obtained by Transient Absorption Spectroscopy

compound	time constant/ns			
	DCM		DMF	
	τ_P_	τ_D_	τ_P_	τ_D_
2Cz-BP^a^	4.3 ± 0.2	530 ± 23	6.9 ± 0.3	2085 ± 210
2tCz-BP^a^	8.3 ± 0.2	2430 ± 250	6.3 ± 0.4	1800 ± 890
2PTZ-BP^a^	0.294 ± 0.020	/	0.170 ± 0.027	/
2PXZ-BP^b^	0.350 ± 0.004	/	0.046 ± 0.001	/
4DP-IPN^b^	2.9 ± 0.1	2720 ± 100	3.0 ± 0.1	3940 ± 540

a 360 nm pump wavelength.

b 425 nm pump wavelength.

### 3.3 Transient Absorption Spectroscopy

**Figure 4:**
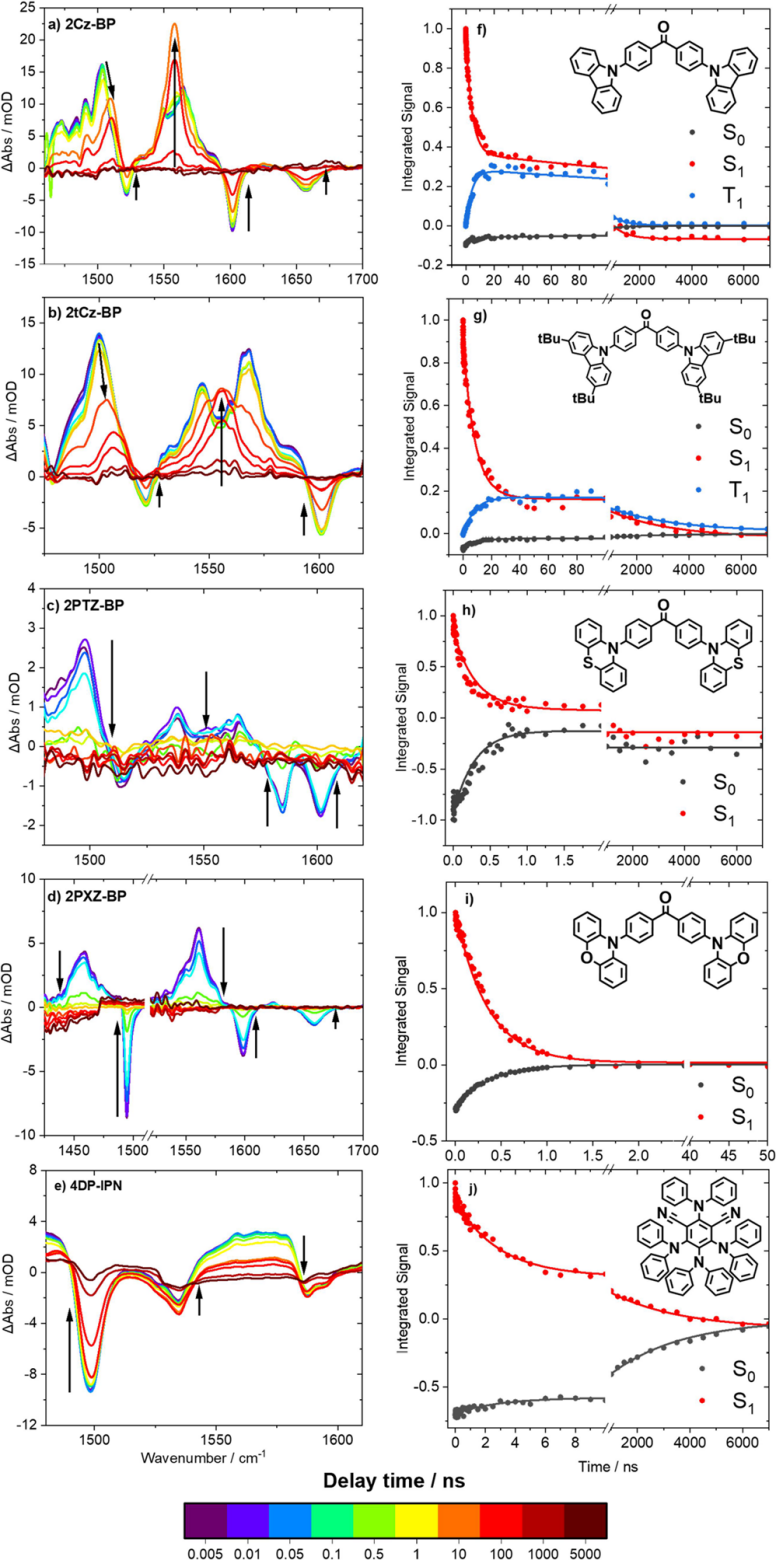
Transient vibrational absorption spectra obtained for time delays from 5 ps to 8 μs for solutions of (a) 2Cz-BP, (b) 2tCz-BP, (c) 2PTZ-BP, (d) 2PXZ-BP, and (e) 4DP-IPN in DCM. Spectra were obtained using a 360 nm UV pump pulse for 2Cz-BP, 2tCz-BP, and 2PTZ-BP (a–c) and a 425 nm pump pulse for 2PXZ-BP and 4DP-IPN (d,e). Spectra are colored to indicate the delay time of the broadband IR probe pulse, and black arrows show the directions of changes of band intensity with time. (f–j) Kinetic traces for the photocatalysts obtained from TVAS measurements in DCM. Solid lines are global exponential fits to data points (closed circles). Fitted exponential time constants are listed in Table 2.

Transient vibrational absorption spectra for each of the five OPCs in DCM are presented in Figure 4. The spectra cover the 1400–1700 cm^–1^ range to observe excited-state absorption (ESA) bands attributed to aromatic ring modes (typically 1400–1600 cm^–1^) as well as depletion of the ground state population by photoexcitation, giving ground state bleach (GSB) features. There are no interfering vibrational absorption bands attributed to DCM in the selected viewing window. In contrast, the use of DMF significantly restricts the IR viewing window because of strong solvent absorption bands. For this reason, spectra acquired in DMF, examples of which are shown in Supporting Information (S5.3), are restricted to the 1530–1610 cm^–1^ range.

In DCM solution, the early time TVA spectra for the carbazole-containing species, 2Cz-BP and 2tCz-BP, each exhibit two broad ESA bands, one with an absorption maximum around 1505 or 1500 cm^–1^ respectively and the other centered around 1560 cm^–1^. We assign these broad ESA features to the vibrationally relaxed S_1_ state because there is no evidence of vibrational cooling from the narrowing of the ESA profile or shifting of the absorption maxima. With increasing time delay, the intensities of the ESA bands decay because of population loss from the S_1_ states. Concurrent growths of new ESA features centered around 1560 cm^–1^ for each compound overlap the declining early time absorptions. Several GSB features are also present in the spectra, the most intense of which lies at approximately 1600 cm^–1^ for both compounds. Analysis of GSB recovery is useful for understanding the dynamics in these systems because the rates and fractional recoveries quantify the time scales and the extents of repopulation of the ground state. The relative contributions of prompt and delayed fluorescence to the recovery of the ground state can therefore be evaluated, bearing in mind that nonradiative relaxation channels might also contribute. Initial and partial recovery of the GSB bands is observed over about 10 ns, indicating direct S_1_ to S_0_ relaxation. As the system evolves further over microsecond time scales, the overlapping transient ESA features decay to the baseline, and GSB recovery is complete.

Table 2 presents kinetic data derived from TVAS measurements for the five OPCs in two solvents. These data were extracted by decomposing the experimental TVAS spectra by using fits to a series of Gaussian functions representative of individual transient ESA or GSB features. In addition, an early time spectrum was used as a basis function to fit the overall relaxation of the excited state. This spectral decomposition used the KOALA software package,^67^ with examples provided in Supporting Information (S5.2). Time constants were obtained by fitting exponential functions to the time-dependent integrated band intensities of the various components of the decomposed spectra, as illustrated in Figure 4.

Kinetic data for the relaxation of the photoexcited 2Cz-BP and 2tCz-BP observed by TVAS were fitted to biexponential decay functions, yielding short (sub 10 ns) and long (microsecond) components of relaxation in both DCM and DMF solutions. A relaxation component on the order of a few picoseconds was also observed using TEAS, with fitted time constants available in the Supporting Information (S6). The kinetic analysis of the ESA features assigned to the S_1_ state provides insights into the temporal evolution of the excited-state population which can be compared with the S_1_ fluorescence lifetimes obtained using TCSPC. For the carbazole-type catalysts in DMF, the smaller time constants of approximately 7.0 ns (hereafter referred to as the prompt component, τ_P_) describe the initial decay of the S_1_ ESA features, partial recovery of the GSB features, and the growth of new, overlapping ESA bands. Comparison of τ_P_ with prompt fluorescence lifetimes obtained via TCSPC (τ_PF_) shows good agreement between the two methods and provides evidence that the prompt component of excited-state relaxation consists, in part, of direct radiative decay from the S_1_ state.

The early time partial recoveries of the GSB features are consistent with the direct repopulation of the ground state by prompt fluorescence emission. The incomplete GSB recovery highlights that the remainder of the S_1_-state population must instead be redistributed to another excited state. For this reason, we assign the ESA features that grow with the same time constant to the population of the T_1_ excited state. This ISC into the triplet manifold also occurs on sub-10 ns time scales. Examination of the longer time constant for GSB recovery (hereafter referred to as the delayed component, τ_D_) reveals that the T_1_-state relaxation occurs on microsecond time scales, giving complete recovery of the ground electronic state population. Delayed TCSPC measurements show that this is, at least in part, a radiative process, with τ_DF_ = (3.26 ± 0.36) μs and (7.9 ± 1.3) μs, for 2Cz-BP and 2tCz-BP, respectively, in DMF. Although the delayed components measured using TVAS (τ_D_ = 2.09 and 1.80 μs for 2Cz-BP and 2tCz-BP) differ from those measured by TCSPC, the time constants determined by the two methods are the same order of magnitude, and discrepancies in the values can be accounted for by more effective exclusion of oxygen in TCSPC measurements compared to TVAS measurements. Therefore, the likely pathway for the few-microsecond population relaxation is by RISC to repopulate the S_1_ state, followed by delayed fluorescence.

As the spectral decompositions summarized in Supporting Information (Section S5) show, the observed ESA features assigned to S_1_-state population decay with time components corresponding to both the prompt and delayed relaxation of the excited-state populations, without changes to the spectral band profiles, confirming that these time-components are associated with the same species. The relaxation time scales match the time constants extracted from our TCSPC measurements, indicating that both prompt and delayed fluorescence are from the excited state of a single species. This behavior is consistent with expectations for a TADF emitter.

By comparing the amplitudes of the prompt and delayed components of GSB recovery kinetics,^31^ quantum yields of triplet formation (Φ_T_) can be directly deduced from TVAS measurements. In DCM, Φ_T_ = 0.62 ± 0.16 for 2Cz-BP and 0.33 ± 0.11 for 2tCz-BP, reducing to 0.21 ± 0.15 and 0.17 ± 0.05 for 2Cz-BP and 2tCz-BP, respectively in DMF. A summary of all derived values is presented in S5.5.

In the TVAS data for 4DP-IPN (Figure 4e), the most intense feature is a GSB at 1500 cm^–1^, with further GSB features of lower intensity also observed around 1535 and 1590 cm^–1^. A broad ESA feature over the range of 1540–1585 cm^–1^ is assigned to the S_1_ state populated by ultrafast IC from the S_2_ state. As was noted above for the carbazole-containing species, the same ESA feature is present over prompt and delayed time scales, confirming repopulation of the S_1_ state via RISC. The kinetics of photoexcited 4DP-IPN relaxation in DCM and DMF are each well described using a global biexponential decay function and yield short (τ_P_*∼*3 ns) and long (τ_D_*∼* 3–4 μs) components. During the shorter-lifetime decay, the ESA bands show a larger proportional decrease from their maximum intensity compared to the recovery of the GSB bands. For example, the broad ESA band centered around 1565 cm^–1^ decreases in intensity by approximately 70% over 5 ns, compared to an approximate 15% recovery of the 1500 cm^–1^ GSB feature over the same period for a solution in DCM. This difference indicates that only a small fraction of the initial S_1_ state population (15%) is directly returning to the ground state, with the remainder most likely undergoing ISC. The τ_P_ and τ_PF_ lifetimes determined via TVAS and TCSPC agree well (see Tables 1 and 2), showing that the partial repopulation of the ground state has a radiative component. TCSPC measurements for 4DP-IPN over extended time delays (Table 1) reveal that the compound also has a long-lived fluorescence component. Examination of the TVAS data at later times shows that the S_1_ ESA feature recovers completely to the baseline, whereas the GSB features exhibit 90% recovery with a τ_D_ time constant of approximately 4 μs. The TCSPC-determined lifetime for delayed fluorescence is an order of magnitude larger than the *τ*_*D*_ time constant observed via TVAS, which we again attribute to more effective exclusion of dissolved oxygen in the TCSPC measurements. Nevertheless, these data indicate that 4DP-IPN exhibits a second radiative component of relaxation that can be temporally separated from the prompt fluorescence. Although there is no direct spectroscopic evidence for triplet formation in this system, such behavior is consistent with RISC and TADF emission. In this system, Φ_T_ = 0.83 ± 0.02 in DCM and increases to 0.99 ± 0.13 in DMF.

1,2,3,5-Tetrakis(carbazol-9-yl)-4,6-dicyanobenzene (4Cz-IPN) structurally resembles 4DP-IPN and is an established TADF emitter^45^ as well as a popular dicyanobenzene-based OPC because of its synthetic versatility.^47−50^ Time constants for relaxation of photoexcited 4Cz-IPN^45,68,69^ are shown in the Supporting Information (S6) for ease of comparison with 4DP-IPN. As we observe here for 4DP-IPN, 4Cz-IPN exhibits two components of excited-state dynamics on short (nanosecond) and longer (microsecond) time scales. The short component is assigned to prompt fluorescence and ISC to the triplet state manifold, whereas the extended, microsecond, component is representative of RISC to repopulate the S_1_ state and subsequent delayed fluorescence. Significantly, the τ_P_ and τ_D_ time constants extracted for 4DP-IPN in this study are similar in magnitude to the corresponding values for 4Cz-IPN obtained from fluorescence lifetime measurements.^68^ Furthermore, recent TVAS measurements of photoexcited 4Cz-IPN from our laboratory,^69^ recorded in the 1450–1540 cm^–1^ wavenumber region, closely resemble those for 4DP-IPN presented here. This close correspondence is significant because neither the 4Cz-IPN nor 4DP-IPN presents any direct spectroscopic evidence of triplet formation in our TCSPC or TVAS measurements, although 4Cz-IPN is widely accepted to be a TADF emitter. As such, it is reasonable to conclude that the close molecular analogue 4DP-IPN also behaves as a TADF emitter.

Figure 4 includes TVAS data for the luminous butterflies, 2PTZ-BP and 2PXZ-BP. Spectra for 2PXZ-BP are presented over a larger wavenumber range than for 2PTZ-BP and exhibit three GSB features centered around 1495, 1600, and 1660 cm^–1^. In addition, two broad ESAs centered around 1460 and 1560 cm^–1^ are assigned to the S_1_ state populated when the long-wavelength ICT band is excited at 425 nm. The spectra of 2PTZ-BP show GSB features at 1585 and 1600 cm^–1^, in addition to a broad ESA centered around 1495 cm^–1^, and a low-intensity ESA band spanning the range 1525–1570 cm^–1^. Despite exciting the S_0_ → S_2_ electronic transition at 360 nm in this compound, the ESA bands correspond to absorptions from the S_1_ state following prompt S_2_ to S_1_ IC on ultrafast time scales not observed by the TVAS experiment. The dynamics of the two photoexcited luminous butterfly OPCs are consistent, with both showing complete GSB recovery and ESA decay to the baseline on subnanosecond time scales. Kinetic analysis of 2PTZ-BP and 2PXZ-BP using monoexponential fits to the decaying band intensities yields subnanosecond lifetimes in both solvents, as reported in Table 2. For example, in DCM, the lifetimes of the excited states are (294 ± 20) and (350 ± 4) ps for 2PTZ-BP and 2PXZ-BP, respectively.

TVAS and TCSPC data for 2PTZ-BP and 2PXZ-BP in DMF show some discrepancies. The TVAS data display monoexponential S_1_ state relaxation with time constants of (170 ± 27) ps and (46 ± 1) ps, whereas the TCSPC data are divided into prompt fluorescence (with a <250 ps decay component and a nanosecond decay component) and delayed fluorescence. The time constants for prompt fluorescence are <250 ps (IRF limited) and (0.88 ± 0.04) ns for 2PTZ-BP and <250 ps (IRF limited) and (1.07 ± 0.04) ns for 2PXZ-BP. For both catalysts, the delayed fluorescence time constant is approximately 300 ns. Both 2PXZ-BP and 2PTZ-BP luminous butterfly species have been reported to be excellent TADF emitters in thin films,^42,55,66^ consistent with the dual lifetime behavior observed by TCSPC measurements of fluorescence emission. However, our TVAS data provide no spectroscopic evidence of triplet formation, and steady-state fluorescence spectra of 2PTZ-BP in 100% DMF (section 3.4) show no substantial fluorescence emission in solution, which is required of a TADF emitter. The absence of fluorescence in the steady-state measurements is consistent with a sub-nanosecond prompt fluorescence lifetime caused by nonradiative S_1_-state quenching and hence a small fluorescence quantum yield.

**Figure 5:**
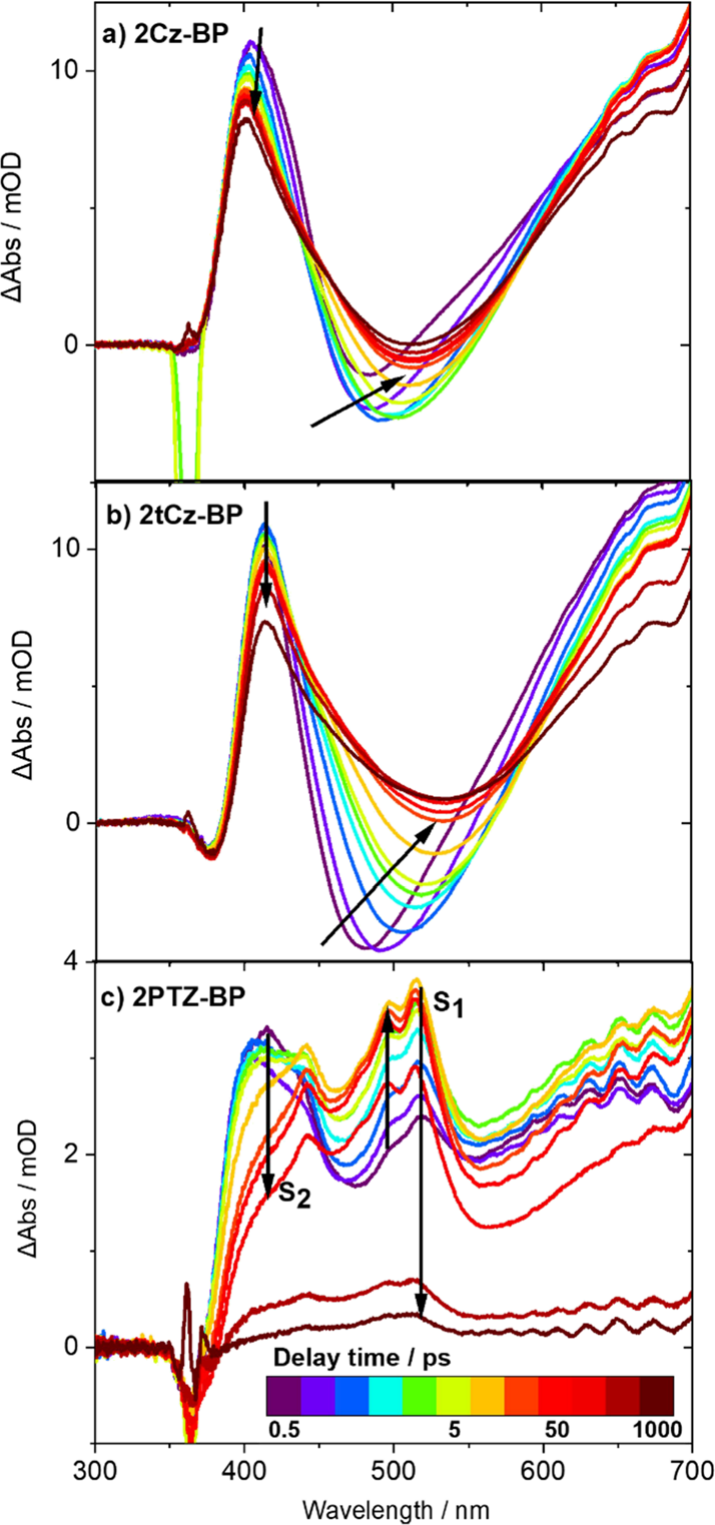
Transient electronic absorption spectra for three organic photocatalysts excited by absorption at 360 nm: (a) 2Cz-BP and (b) 2tCz-BP in DMF; and (c) 2PTZ-BP in DCM. Spectra are presented at time delays from 0.5 ps to a maximum time delay of 1 ns. Black arrows indicate the directions of the evolution of transient absorption features with increasing delay times. Sharp cutoffs in the ESA bands at wavelengths below 370 nm are caused by pump-beam scatter and the decrease in intensity of the white-light continuum used for TEAS.

To understand better the early time behavior of the luminous butterfly OPCs, transient electronic absorption spectroscopy experiments were used to observe the dynamics on time scales from 300 fs to 1.3 ns. Examples of the TEAS measurements are shown in Figure 5 for 2Cz-BP and 2tCz-BP in DMF and for 2PTZ-BP in DCM. Despite substantial overlap of spectral features, decomposition of the TEAS spectra for 2PTZ-BP (SI Section S5.1) reveals contributions from two electronically excited states: a sharp S_2_ ESA band centered around 410 nm decays by ultrafast IC with a time constant τ_UF_ = 4.7 ps and an S_1_ ESA band with a double-peaked maximum centered around 508 nm grows in intensity on the same time scale, before decaying with an exponential lifetime of 275 ps. This latter time constant agrees well with the τ_P_ = 294 ps lifetime for S_1_ decay extracted from TVAS measurements (Table 2). The subnanosecond S_1_ prompt lifetime determined by both TEAS and TVAS measurements is consistent with the short component of prompt fluorescence decay that is within the TCSPC IRF (Table 1) but is not representative of the nanosecond component of prompt S_1_ fluorescence decay. Further comparisons are drawn between these measurements in Section 3.4.

TEAS spectra for 2Cz-BP and 2tCz-BP in DMF are presented in Figure 5a,b. The spectral profiles for these two carbazole-containing OPCs are similar, exhibiting a short-wavelength ESA (centered around 400 and 413 nm for 2Cz-BP and 2tCz-BP, respectively) as well as a broad stimulated emission (SE) feature, which was visible to the eye as a green glow from the UV-excited sample. The SE features shift to longer wavelengths (by approximately 30 nm in 2Cz-BP and 60 nm in 2tCz-BP) at short time delays of a few picoseconds. For 2Cz-BP, the partially overlapping ESA band narrows and shifts to a shorter wavelength on the same time scale. These changes to the spectra reflect vibrational cooling (by energy transfer to the solvent) or structural relaxation in the S_1_ state. Although changes to the S_1_ band profile in 2tCz-BP are less pronounced, the shift of the SE feature is also consistent with relaxation in the S_1_ state, and ultrafast time constants, τ_UF_, extracted from these spectra show that this relaxation occurs on time scales of approximately 3 ps. Persistence of the S_1_ ESA bands beyond the 1 ns measurement limit of our TEAS experiment is consistent with the ∼7 ns τ_PF_ and τ_P_ values reported in Tables 1 and 2, respectively.

### 3.4 Effects of OPC Aggregation on TADF Behavior

In Section 3.3, we highlight a discrepancy between *τ*_*P*_ values obtained for 2PTZ-BP and 2PXZ-BP using TEAS or TVAS compared with τ_PF_ values measured using TCSPC. These discrepancies are evident from comparison of the reported time constants in Tables 1 and 2. In transient absorption spectroscopy experiments, the excited-state dynamics are described using a monoexponential decay function, giving prompt excited-state lifetimes of τ_P_ = (170 ± 27) and (46 ± 1) ps in DMF for 2PTZ-BP and 2PXZ-BP, respectively. However, fluorescence lifetimes obtained from TCSPC experiments are better described in terms of prompt and delayed fluorescence decay traces. For 2PTZ-BP, the prompt fluorescence has two decay components with time constants of <250 ps (IRF limited) and (0.88 ± 0.04) ns and delayed fluorescence with τ_DF_ = (314 ± 10) ns. For 2PXZ-BP, the corresponding values are <250 ps (IRF limited) and (1.07 ± 0.04) ns for prompt fluorescence, while τ_DF_ = (304 ± 20) ns. In both cases, we recognize that the IRF limited signal is enhanced by Raman scattering, as discussed earlier. We propose that the contrasting relaxation kinetics obtained from the transient absorption and fluorescence emission measurements are a consequence of different experimental sensitivities to monomers or a small fraction of aggregated OPCs, in the studied solutions.

Aggregation-induced emission luminogens (AIEgens) are species that exhibit enhanced fluorescence lifetimes on aggregation and have been explored for technologies where nonradiative quenching of excited states on aggregation is undesirable, particularly in thin films.^70^ A previous study of the delayed fluorescence properties of the luminous butterfly compounds 2PTZ-BP and 2PXZ-BP for biological fluorescence lifetime imaging identified a significant increase in the photoluminescence quantum yield (PLQY) and subsequently TADF emission, in films compared to tetrahydrofuran (THF) solution.^66^ X-ray crystallography of 2PXZ-BP confirmed the twisted structures that these compounds adopt and guided the conclusion that in aggregates, close π–π stacking is restricted by the twisted geometry, therefore inhibiting nonradiative quenching pathways. Furthermore, restrictions to the intramolecular motion about the N*–*Ph bond of the donor–acceptor units, and to the puckering of phenothiazine rings by the constrained geometries, promoted radiative emission in aggregates by blocking nonradiative decay routes accessed via geometric changes.^71^ Recognizing that intramolecular motion facilitates rapid nonradiative decay, we might not expect to observe fluorescence of these luminous butterfly OPCs in dilute solution because the nonaggregated molecules are conformationally more flexible than in thin films. We propose that our TEAS and TVAS measurements, which do not require the sample to fluoresce, report the photochemical dynamics of these solvated monomeric OPCs. By measuring emitted photons from fluorescent species, TCSPC is instead likely to be sensitive to low concentrations of aggregated OPCs present even in dilute solutions while being less sensitive to the more weakly emissive monomers. The different *τ*_*P*_ values obtained by transient absorption and TCSPC spectroscopies therefore reflect preferential detection of nonaggregated and aggregated 2PTZ-BP and 2PXZ-BP, respectively, and the different relaxation pathways for the photoexcited molecules in these two forms.

**Figure 6:**
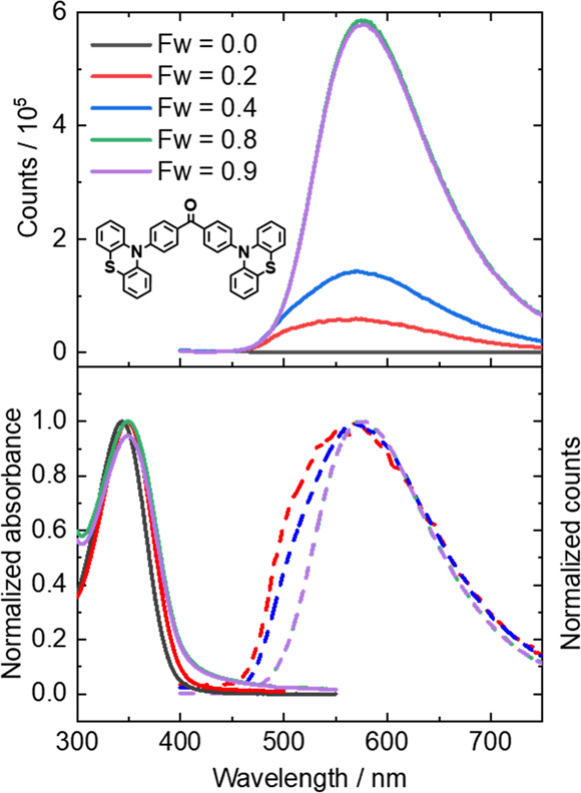
Steady-state fluorescence and absorption spectra for 2PTZ-BP in mixed DMF–water solutions, with water fractions of *F*_w_ varying from 0.0 to 0.9. Top: fluorescence spectra of 2PTZ-BP obtained using an excitation wavelength of 360 nm. Bottom: normalized UV–visible absorption (solid lines) and fluorescence spectra (dashed lines).

To investigate the proposed effect of aggregation on PLQY values, steady-state UV–visible absorption spectra and fluorescence spectra were recorded for 2PTZ-BP solutions in DMF: water mixtures. The degree of aggregation was expected to increase with greater volume fractions of water (*F*_w_) in the mixed solution because 2PTZ-BP is soluble in DMF but not in water. The selective partitioning of the solute OPC molecules into the DMF fraction increases the effective concentration of 2PTZ-BP in DMF as *F*_w_ increases without changing the overall 2PTZ-BP concentration of the solution. Example absorption and fluorescence spectra are shown in Figure 6.

The fluorescence spectra for the 2PTZ-BP solutions show that increasing the volume fraction of water to promote aggregation substantially increases the emission intensity. A bathochromic shift in the UV–visible absorption and fluorescence maxima is also observed as the *F*_w_ increases. Furthermore, in solutions favoring the aggregated states (*F*_w_ = 0.8 and 0.9), the absorption bands become broader, developing tails at longer wavelengths, which we also attribute to aggregation. For roughly equal volumes of DMF and water, a suspension was produced which scattered the incident radiation in the fluorescence or UV–visible spectrometers. For this reason, spectra for solutions with *F*_w_ = 0.4 and 0.6 are omitted in Figure 6.

Aggregation-induced emission of 2PTZ-BP provides a plausible explanation for the observed discrepancies in relaxation time constants extracted from transient absorption and TCSPC measurements for this OPC. In dilute solution, the luminous butterfly OPCs favor monomeric solvated forms and are not restricted conformationally by aggregation. Intramolecular motion in the S_1_ state therefore facilitates rapid nonradiative relaxation to the electronic ground state, with little competing radiative (fluorescence) decay. The TCSPC measurements are sensitive only to emitting species and therefore favor the radiative decay from the small fraction of OPC molecules in the dilute solution that are present in dimers or higher aggregates. The TCSPC is less sensitive to the monomers in solution because their fluorescence quantum yields are small. In environments where aggregation occurs (e.g., as is required for thin-film optical displays), intermolecular interactions between adjacent molecules in the aggregates restrict the free molecular motion of the solvated monomers, thereby inhibiting ultrafast nonradiative IC to the ground state. In such systems, fluorescence decay routes become competitive with ISC channels and the dynamics are better described by a two-component kinetic model, in which prompt fluorescence and ISC occur, followed by RISC and delayed fluorescence. However, a comparison of prompt and delayed fluorescence amplitudes measured by time-resolved fluorescence indicates that Φ_T_ < 0.05 for this class of OPC in both DCM and DMF, consistent with our TVAS determinations in Table S9, indicating that ISC is an inefficient process in solution. Consequently, 2PXZ-BP and 2PTZ-BP behave as TADF emitters in thin films but not in bulk solution.

### 3.5 Summary

**Figure 7:**
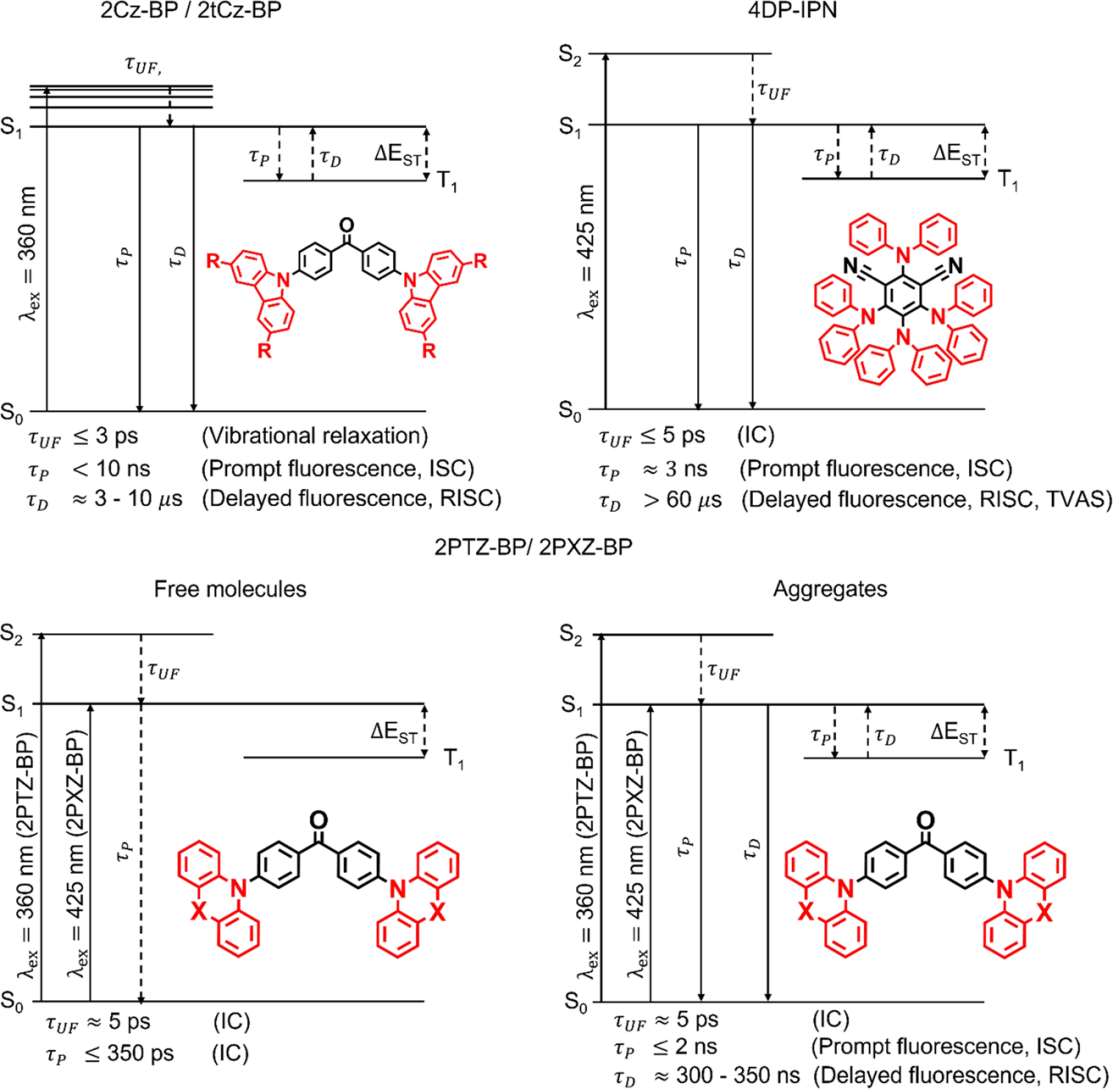
Summary of the proposed dynamics for each class of photocatalyst with associated time constants. Radiative pathways are represented by solid lines and nonradiative pathways by dashed lines. IC will contribute to S_1_ → S_0_ repopulation in competition with radiative decay but is omitted from the diagrams to highlight TADF behavior.

The proposed excited-state dynamics for each class of photocatalyst studied here are summarized in Figure 7. All five compounds investigated can behave as TADF emitters, but the PLQY values depend on the class of OPC and the molecular environment. For the carbazole-containing catalysts 2Cz-BP and 2tCz-BP, TVAS reveals a two-component relaxation pathway, in which prompt fluorescence decay to the ground electronic state is competitive with ISC into the triplet manifold. The formation of a triplet excited state is supported by the observed growth of an overlapping ESA band during the τ_P_ lifetime, and the competitive radiative decay from S_1_ is confirmed by the TVAS and TCSPC data. Similar dynamics are proposed for 4DP-IPN, informed by prompt and delayed components of emission in TCSPC fluorescence measurements. Relaxation of the excited-state population back to S_0_ on two time scales is also observed through the partial recovery of GSB features in TVAS measurements. The assignment of 4DP-IPN as a TADF emitter is consistent with the known behavior of the dicyanobenzene analogue 4Cz-IPN. In both cases, redistribution of the S_1_-state population occurs via ISC into the triplet manifold.^45^

The photodynamic behavior of 2PTZ-BP and 2PXZ-BP is found to be more complicated and sensitive to the experimental conditions. In environments that facilitate aggregation, steady-state and TCSPC fluorescence spectroscopy show 2PTZ-BP and 2PXZ-BP to be emissive because conformational restrictions imposed by aggregation inhibit ultrafast relaxation pathways in the excited states. As a result, fluorescence becomes a competitive deactivation pathway in aggregates containing photoexcited molecules, and both 2PTZ-BP and 2PXZ-BP can exhibit TADF behavior. However, in dilute solutions, the monomeric, solvated OPCs are not conformationally restricted, so intramolecular motion enables the photoexcited S_1_ state molecules to return to the ground state via nonradiative decay pathways on time scales of a few hundred picoseconds. Therefore, ISC, RISC, and TADF emissions are not observed.

Understanding the fundamental dynamics and potential energy landscapes of TADF emitters should inform design improvements to OPCs such that TADF behavior is either enhanced or suppressed, as required by applications in photocatalyzed chemical synthesis. For efficient TADF emission, the singlet–triplet energy gap Δ*E*_ST_ should be small, and twisted molecular architectures are an efficient way to achieve this objective.^39,42,54,65^ Many effective OPCs have low-lying ICT states, adopting a D–A type scaffold that might allow targeted modification of donor and acceptor moieties to fine-tune the excited-state energies.

The ISC and RISC pathways may not occur directly between the S_1_ and T_1_ states because guidance from El Sayed’s rule indicates the need for a change in orbital character.^72^ Instead, ISC pathways may require population to transfer from S_1_ to T_2_ (or a higher-lying triplet state) followed by T_2_ → T_1_ internal conversion.^43,68,73,74^ Therefore, focusing on the effect that the S_1_–T_1_ energy gap, Δ*E*_ST_, has on the RISC time scales overlooks the importance of the relative energies of possible intermediary states such as T_2_ in the nonadiabatic photodynamics. For ISC to occur via T_2_, the excited-state geometry of an OPC must distort to a structure where the S_1_ and T_2_ states are degenerate, allowing transfer of the excited-state population between the singlet and triplet manifolds. The structural changes lead to an activation energy (*E*_a,ISC_) for ISC. Similarly, for RISC to occur via this intermediate T_2_ triplet state, *E*_a,RISC_ is determined by the difference in energy between the minimum of the T_1_ potential and the intersection of the T_2_ and S_1_ potentials. Because *E*_a,RISC_ = Δ*E*_ST_ + *E*_a,ISC_, the rate of RISC, and therefore TADF behavior, depends not only on the S_1_–T_1_ energy gap Δ*E*_ST_ but also on other factors such as the energy of the S_1_/T_2_ intersection. Consequently, when designing TADF emitters, including for applications as organic photocatalysts, it is necessary to consider the effects of both Δ*E*_ST_ and *E*_a,ISC_.

## 4 Conclusions

Both time-resolved fluorescence spectroscopy and transient absorption spectroscopy methods have been used to characterize the excited-state dynamics of three classes of D–A type molecular architectures recently tested as organic photoredox catalysts for ATRP. Two of these classes consist of a central benzophenone acceptor motif appended either with carbazole or phenoxazine/phenothiazine derivatives (luminous butterflies), whereas the third class is a 4,6-dicyanobenzene core bound to four NPh_2_ donor moieties. Measurements made from subpicosecond to microsecond time scales provide a comprehensive picture of RISC and TADF behavior in these candidate OPCs for photoredox applications. Transient absorption spectroscopies show evidence of RISC and TADF emission for 2Cz-BP, 2tCz-BP, and 4DP-IPN in two solvents through biexponential GSB recovery kinetics as well as direct observation of ESA bands assigned to the T_1_ state in TVA spectra for carbazole-type species. Biexponential fluorescence decay on nanosecond time scales observed via time-resolved fluorescence spectroscopy also reveals TADF emission in all the species studied.

For one class of luminous butterflies (2PXZ-BP and 2PTZ-BP with phenoxazine/phenothiazine donor units), we see evidence of RISC in TCSPC experiments but not in transient absorption spectroscopy experiments. The differences are attributed to differing sensitivities of the techniques to monomers or aggregates in solution. Transient absorption spectra are dominated by a majority species of monomers in solution, which are only weakly fluorescent, and observe rapid nonradiative excited-state relaxation of these monomers on sub-nanosecond time scales. However, TCSPC is more sensitive to the small fraction of molecular aggregates (dimers or higher order) which fluoresce more strongly than the monomers, meaning that radiative relaxation dynamics of the S_1_ states of aggregated molecules are observed. As such, the 2PTX-BP and 2PTZ-BP OPCs behave as AIEgens.

The potential for TADF emitters to be candidate OPCs for chemical and materials synthesis has been investigated by Kwon and co-workers.^20,57,75^ The outcomes indicated the importance of triplet excited states in efficient photoinduced electron transfer processes and highlighted the role of ICT states in facilitating triplet generation in the organic chromophores. Organocatalyzed ATRP studies, using the five OPCs investigated here, showed a correlation between OPC triplet state quantum yields, monomer conversion, and initiator efficiency.^57^ They also revealed much poorer ATRP yields using 2PXZ-BP and 2PTZ-BP photocatalysis (see Table S12 in the Supporting Information for a summary). High Φ_T_ values were deemed more important for organocatalyzed oxidative quenching cycles than the excited-state oxidation potentials of the photocatalysts. Moreover, triplet states in reductively quenched cyanoarene OPCs used for radical anion-mediated photoredox catalysis inhibit back electron transfer processes. When used as OPCs, TADF emitters have the benefit of similar thermodynamic driving forces, and hence Marcus-theory rates, for electron transfer reactions from their T_1_ as their S_1_ states because of their energetic proximity.^76^ To add to these mechanistic insights, the quantitative information about RISC and TADF propensities, excited-state lifetimes, and triplet quantum yields presented here accounts for the poor O-ATRP performance of 2PXZ-BP and 2PTZ-BP, and it will inform the design of new OPCs intended for photoredox catalysis.

Direct observation of triplet state formation in high quantum yields for 2Cz-BP and 2tCz-BP using TVAS supports the conclusion that such states play a key role in photoredox catalysis, and the results of this investigation show efficient TADF emission from these twisted D–A molecules. As advocated by Adachi and co-workers,^43^ when designing molecules to optimize TADF behavior, the role of the intermediate T_2_ state on ISC/RISC dynamics must be considered as well as the S_1_–T_1_ energy gap. Using a twisted molecular architecture to achieve ICT excited states, it is feasible to modify donor and acceptor moieties independently to adjust the relative energies of the S_1_, T_1_, and T_2_ states of the OPCs, allowing directed control of TADF behavior and hence the performance of the OPC in photoredox catalysis.

## Data Availability

Data are available at the University of Bristol data repository, data.bris, at 10.5523/bris.2pbd4kxoiyhbg28ustgeyh3rxn.

## Supporting Information Available

Additional method details, synthesis of compounds, ^1^H NMR spectra for OPCs, FTIR spectra for OPCs in DCM, frequency calculations and additional NTO projections for OPCs calculated using DFT, prompt and delayed time-resolved fluorescence traces in DMF, spectral decomposition of TEA and TVA data, TVAS data and kinetic modeling for OPCs in DMF, calculations for triplet quantum yields, ultrafast time constants for OPCs, and reference time constants for 4Cz-IPN (PDF)
