# Supplementary material for: Characterization of the Reversible Intersystem Crossing Dynamics of Organic Photocatalysts Using Transient Absorption Spectroscopy and Time-Resolved Fluorescence Spectroscopy
Source: J Phys Chem A. 2023 Dec 14;127(51):10775–88. doi: 10.1021/acs.jpca.3c04780 (PMC10758116; doi:10.1021/acs.jpca.3c04780)
Supplement: Supplementary file 1 — jp3c04780_si_001.pdf [file jp3c04780_si_001.pdf]

Characterization of the Reversible Intersystem Crossing Dynamics of Organic Photocatalysts using Transient Absorption Spectroscopy and Time-Resolved Fluorescence Spectroscopy

William Whitaker,[a] Igor V. Sazanovich,[c] Yonghwan Kwon,[b] Woojin Jeon,[b] Min Sang Kwon,[b] and Andrew J. Orr-Ewing [a,*]

[a] *School of Chemistry, University of Bristol, Cantock's Close, Bristol BS8 1TS, UK*

[b] *Department of Materials Science and Engineering, Seoul National University, Seoul 08826, Republic of Korea*

[c] *Central Laser Facility, Research Complex at Harwell, Science and Technology Facilities Council, Rutherford Appleton Laboratory, Harwell Oxford, Didcot, Oxfordshire, OX11 0QX, UK*

* Author for correspondence: a.orr-ewing@bristol.ac.uk

Contents **Page**

S1 Methods **S3**

- **S1.1 Spectroscopy** **S3**
- **S1.2 Synthesis** **S5**

S2 Steady State Measurements **S9**

- **S2.1 Experimental Details for Steady State Measurements** **S10**
- **S2.2 Comparison of FTIR Experiments with DFT Calculations** **S10**
- **S2.3 Steady State Photoluminescence Emission Spectroscopy** **S11**

S3 Natural Transition Orbital Diagrams for Key States **S12**

- **S3.1 Carbazoles** **S12**
- **S3.2 2PTZ-BP** **S13**
- **S3.3 4DP-IPN** **S13**

S4 Time-Resolved Fluorescence Traces **S14**

- **S4.1 Time-Resolved Fluorescence Traces for OPCs in DMF and DCM** **S14**
- **S4.2 Emission Wavelength Dependent TCSPC** **S20**
- **S4.3 TCSPC for Aggregates of 2PTZ-BP and 2PXZ-BP** **S24**

S5 Analysis of Transient Absorption Spectra **S25**

- **S5.1 Decomposition of TEA Spectra** **S25**
- **S5.2 Decomposition of TVA Spectra Measured in DCM** **S27**

S5.3 Additional TVAS and Kinetics Measurements in DMF S30

S5.4 Decomposition of TVA Spectra Measured in DMF S31

S5.5 Triplet Quantum Yields S34

S6 Additional Time Constants S35

S7 O-ATRP performance for the organic photocatalysts S36

S1 Methods

S1.1 Spectroscopy

Transient electronic absorption spectroscopy (TEAS) and transient vibrational absorption spectroscopy (TVAS) measurements of the photodynamics of five OPCs in two solvents used ultrafast laser systems at the University of Bristol and the LIFEtime facility at the STFC Rutherford Appleton Laboratory, respectively. The details of each system have been comprehensively described elsewhere.[1-3]

For TEAS experiments at the University of Bristol, the 800-nm output of an amplified pulsed Titanium-sapphire laser (Coherent Legend Elite HE+, 5 W, 1 kHz) was split to generate pump and probe pulses. The wavelength-tuneable UV pump pulses (λ = 360 nm for all samples) were generated from the 800-nm beam using a Coherent OperA Solo optical parametric amplifier (OPA) to give pulse energies of 500 nJ at the sample. An optical delay stage in the 360-nm beam path controlled the time interval between pump and probe laser pulses, and a 500 Hz chopper was used to acquire pump-on and pump-off data in sequential laser shots. A small portion of the 800-nm output from the laser amplifier generated broadband white-light continuum (WLC) probe pulses spanning wavelengths from 350 – 700 nm by focusing into a 3-mm thick CaF_2 window. WLC pulses were recollimated by an off-axis parabolic mirror and focussed into the sample. This setup allowed measurements to be recorded over time delays from ~ 100 fs to 1.3 ns.

For TVAS measurements using the LIFEtime facility, UV pump wavelengths of 360 nm and 425 nm were selected, with an average pulse energy of 300 nJ at the sample. Pairs of ~200 cm^{-1} bandwidth IR probe pulses, offset in central wavenumber but spatially overlapped at the sample, covered the wavenumber range 1400-1800 cm^{-1}. These wavelength-tuneable pump and probe pulses were generated in separate OPAs (Light Conversion Orpheus HP and Orpheus ONE). A combination of an optical delay stage and pulse picking from the dual-amplifier (Light Conversion, Pharos, 15 W, 100 kHz, 260 fs output and Pharos SP, 6W, 100 KHz, 180 fs) laser system covered pump-probe time delays from < 1 ps to several microseconds.

The concentrations of the organic photocatalyst solutions used in TEAS and TVAS measurements were 1.0 – 2.5 mM, with typical concentrations being 1.0 mM (2Cz-BP), 1.25 mM (2tCz-BP), 1.5 mM (2PTZ-BP) and 2.5 mM (2PXZ-BP and 4DP-IPN). Samples were prepared in dichloromethane (DCM, spectroscopy grade, $\geq$ 99.9%) or *N,N*-dimethyl formamide (DMF, spectroscopy grade, $\geq$ 99.9%) and were circulated through a stainless-steel Harrick cell (200 μm path length) fitted with CaF_2 windows using a peristaltic pump. Samples for TVAS were sparged with dry nitrogen gas to reduce the influence of quenching by dissolved oxygen. This sparging was not necessary for TEAS measurements with a maximum delay time of 1.3 ns.

Aggregation experiments for solutions of 2PTZ-BP were conducted using a Thermo Scientific Genesys 10S UV-Vis spectrometer, and an Edinburgh Instruments Spectrofluorometer FS5. In these measurements, 5 mL dilute solutions of 2PTZ-BP were prepared with various DMF to water ratios (by volume), and steady-state UV-Visible absorption spectra and fluorescence spectra (with a 360-nm excitation wavelength) were recorded using a 1 cm quartz cuvette.

Steady-state PL emission spectra at room temperature were obtained with a QuantaMaster 40 UV/vis steady state spectrofluorometer (Photon Technology International Inc.) equipped with a

75W Xe short arc lamp. The emission spectra were corrected for the sensitivity of the photomultiplier tube.

Time-correlated single photon counting (TCSPC) experiments of 10 μM OPC solutions were conducted at Seoul National University to characterize prompt and delayed components of fluorescence. The excitation source was a 377 nm pulsed diode laser (LDH series PicoQuant) of pulse width (FWHM) < 49 ps. Prompt fluorescence decay measurements were carried out by a PicoHarp−300 TCSPC event timer (PicoQuant) with 64 ps time resolution. Soapy water was used to scatter excitation light for measurement of the instrument response function (IRF). In prompt fluorescence measurements, the samples were not sparged to eliminate any contribution of delayed fluorescence to the measurements. Delayed fluorescence measurements were carried out by a NanoHarp−250 TCSPC event timer after degassing dissolved oxygen by sparging for 10 mins with 99.9999% Ar gas. The decay time fitting procedure was carried out using Origin for prompt fluorescence emission, and the Fluofit software (PicoQuant) for delayed fluorescence emission.

S1.2 Synthesis

2Cz-BP: NaH (0.22 g, 60% in mineral oil, 9.16 mmol) was taken in a dried two neck round bottom flask and a solution of carbazole (0.842 g, 5.03 mmol) in anhydrous DMAc (8 ml) was added slowly and allowed to stir for 30 mins at room temperature. To this, a solution of 4,4'-difluorobenzophenone (0.5 g, 2.29 mmol) in anhydrous DMAc (5 ml) was added and the mixture was allowed to stir at 60 °C for 5 h. After cooling to room temperature, the reaction mixture was poured into water and the resulting off white precipitate was filtered. The obtained crude product was then purified via column chromatography using CH_2Cl_2: hexanes as eluent to obtain desired pure product as pale yellow powder (0.86 g, 74%). ^1H NMR (400 MHz, $CDCl_3$): δ 8.20 – 8.14 (m, 8H), 7.83 – 7.77 (d, 4H), 7.59 – 7.53 (d, 4H), 7.49 – 7.43 (t, 4H), 7.37 – 7.31 (t, 4H). NMR data is consistent with reported data.[4]

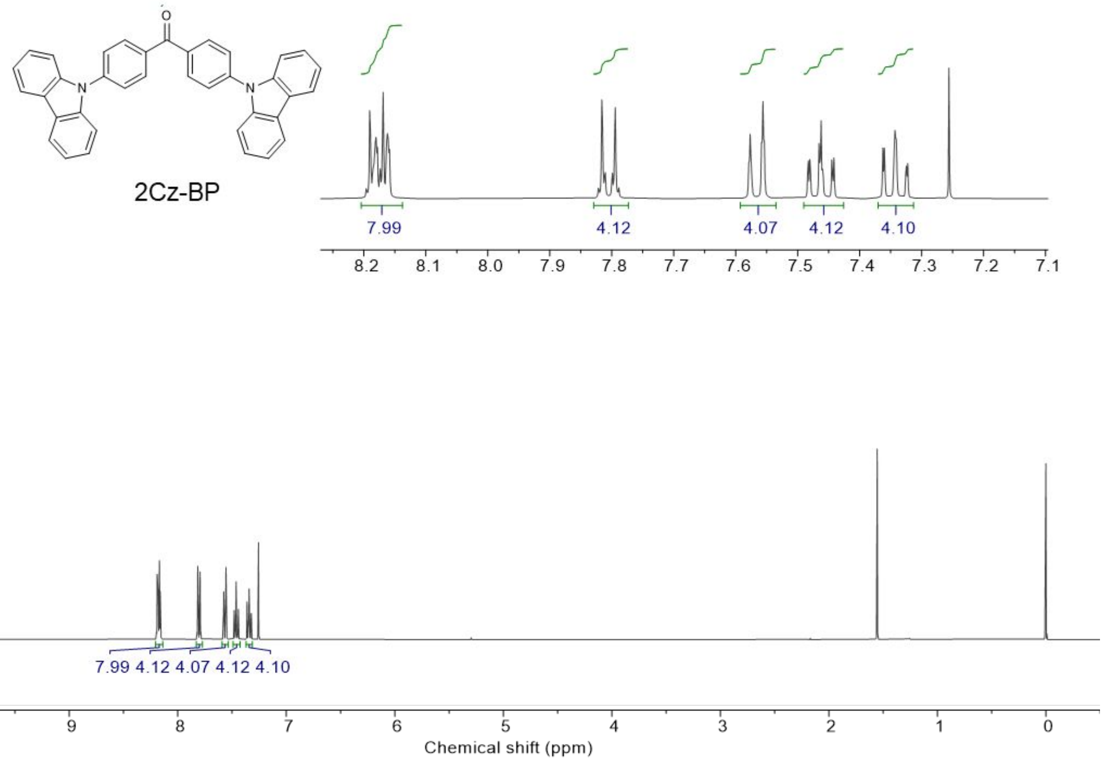

Figure S1 ^1H NMR data of 2Cz-BP at RT (400 MHz, $CDCl_3$).

2tCz-BP: To a mixture of *t*-BuOK (0.090 g, 0.8 mmol), 3,6-di-tert-butyl-9H-carbazole (0.200 g, 0.71 mmol) and 4,4'-difluorobenzophenone (0.062 g, 0.28 mmol), 5 mL anhydrous DMF was added and the reaction mixture was stirred at 90 °C for 10 h. The reaction mixture was allowed to cool to room temperature. Water (20 ml) was added and extracted with $CHCl_3$, followed by drying with sodium sulfate and concentration under reduced pressure. The obtained residue was purified by column chromatography on silica gel using CH_2Cl_2: hexanes (1:3, v/v) as eluent system to give off white solid as a pure product (0.171 g, 81%). ^{1}H NMR (400 MHz, $CDCl_3$): δ 8.18 – 8.11 (m, 8H), 7.81 – 7.76 (d, 4H), 7.51 (d, 8H), 1.48 (s, 36H). NMR data is consistent with reported data.[5]

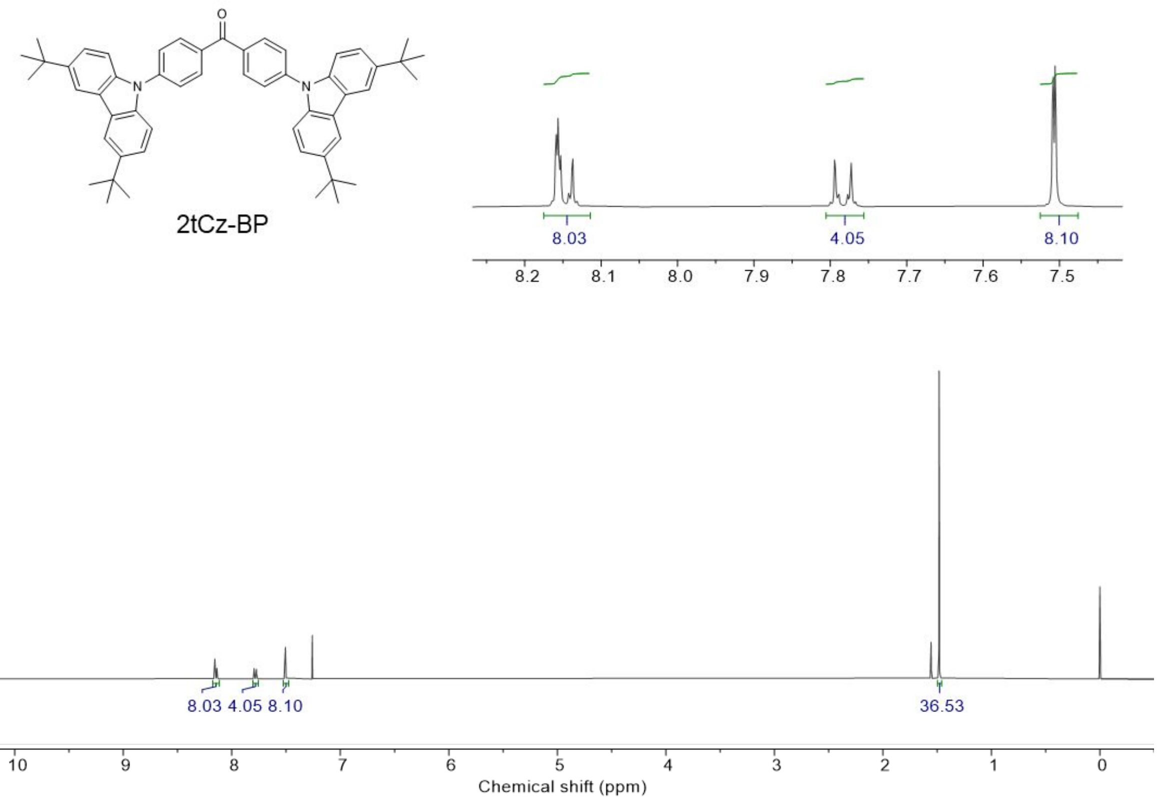

Figure S2 ^{1}H NMR data of 2tCz-BP at RT (400 MHz, $CDCl_3$).

2PTZ-BP: To a suspension of NaH (60% in mineral oil, 0.063 g, 1.57 mmol) in anhydrous DMAc (1 ml) at 0 °C, a solution of phenothiazine (0.250 g, 1.25 mmol) in anhydrous DMAc (2 ml) was added and allowed to stir for 30 mins at room temperature. 4,4'-difluorobenzophenone (0.109 g, 0.50 mmol) in anhydrous DMAc (2ml) was then added dropwise. The resultant solution was stirred overnight at 100 °C. The reaction mixture was cooled to room temperature and quenched with ice cold water (15 ml) before being further diluted with water and extracted with dichloromethane, dried on Na_2SO_4 and concentrated under reduced pressure. The obtained residue was purified by column chromatography on silica gel using CH_2Cl_2: hexanes (1:3, v/v) as eluent system to give off white solid as pure product (0.245 g, 85%). ^{1}H NMR (400 MHz, $CDCl_3$): δ 7.87 – 7.82 (d, 4H), 7.31 – 7.27 (d, 4H), 7.27 – 7.24 (d, 4H), 7.18 – 7.12 (t, 4H), 7.09 – 7.03 (t, 4H), 6.93 (d, 4H). NMR data is consistent with reported data.[6]

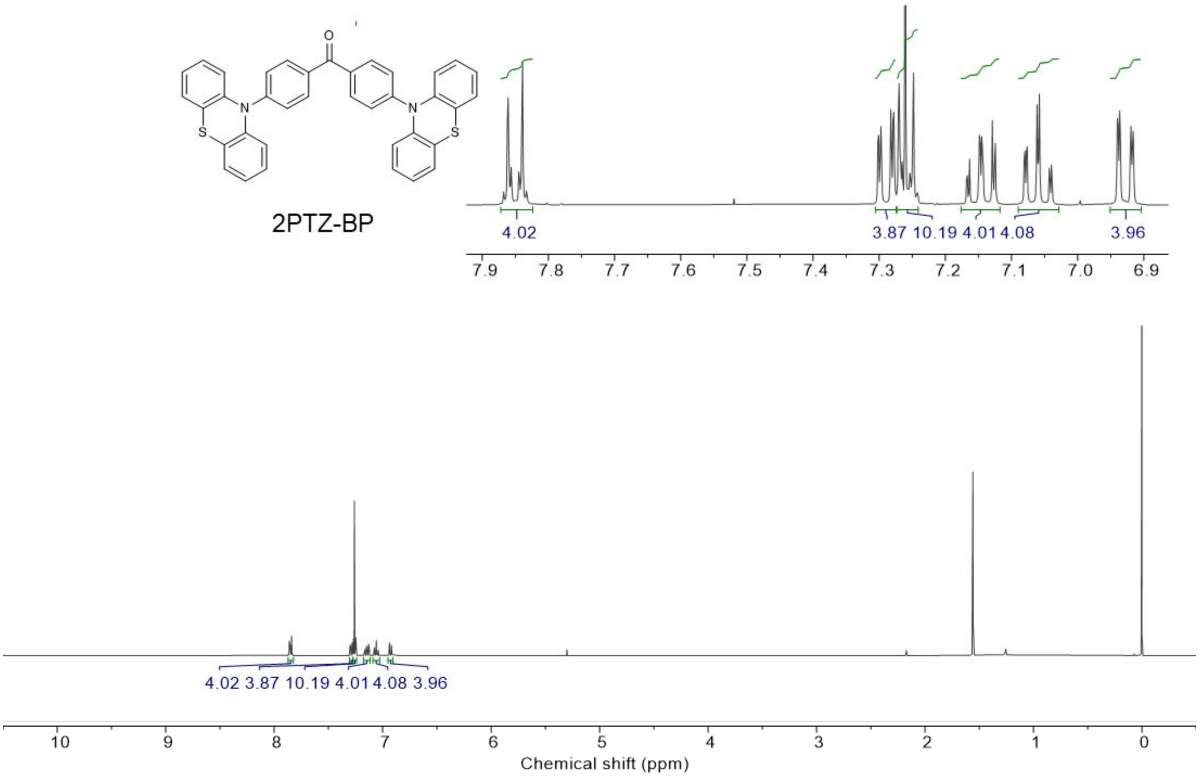

Figure S3 ^{1}H NMR data of 2PTZ-BP at RT (400 MHz, $CDCl_3$).

2PXZ-BP: NaH (0.22 g, 60% in mineral oil, 9.16 mmol) was taken in a dried two neck round bottom flask and a solution of phenoxazine (0.923 g, 5.03 mmol) in anhydrous DMAc (8 ml) was added slowly and allowed to stir for 30 mins at room temperature. To this, a solution of 4,4'-difluorobenzophenone (0.5 g, 2.29 mmol) in anhydrous DMAc (5 ml) was added and the mixture was allowed to stir at 60 °C for 4 h. After cooling, the reaction mixture was poured into water and the resulting yellow precipitate was filtered followed by washing with CH_3OH. The obtained crude product was then recrystallized from $CHCl_3$/MeOH to obtain desired pure product (0.59 g, 55%). ^1H NMR (400 MHz, $CDCl_3$): δ 8.17 – 8.02 (d, 4H), 7.59 – 7.45 (d, 4H), 6.78 – 6.55 (m, 12H), 6.02 (d, 4H). NMR data is consistent with reported data.[7]

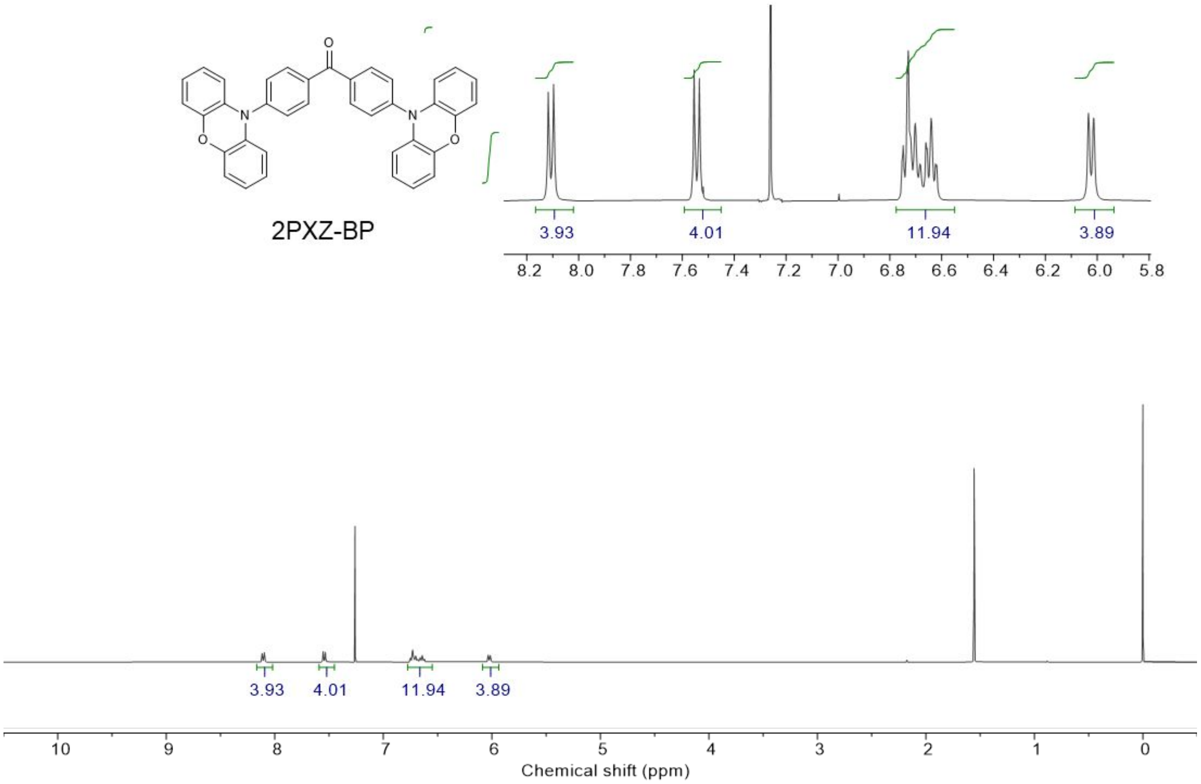

Figure S4 ^1H NMR data of 2PXZ-BP at RT (400 MHz, $CDCl_3$).

4DP-IPN: A solution of NaH (60% in mineral oil, 0.477g, 11.94 mmol) and diphenylamine (1.48 g, 8.75 mmol) in anhydrous DMAc (5 mL) was stirred for 30 mins in ice bath under a nitrogen atmosphere. After 30 mins, tetrafluoroisophthalonitrile (0.4 g, 1.99 mmol) dissolved in DMAc (5 mL) was slowly added to the reaction mixture and stirred further at 100 ^{o}C for 10 h. Afterwards, distilled water (2 mL) was poured into the reaction mixture to quench the excess NaH, and CH_3OH was added to precipitate the crude product which was further purified by column chromatography on silica gel (CH_2Cl_2:hexanes, 2:3 v/v) to give pure product as yellow solid (1.32 g, 83%). ^{1}H NMR (600 MHz, $CDCl_3$): δ 7.29 – 7.24 (t, 4H), 7.10 – 7.05 (t, 12H), 7.02 (t, 2H), 6.93 – 6.89 (t, 4H), 6.87 (t, 4H), 6.72 – 6.67 (m, 10H), 6.57 – 6.53 (d, 4H). NMR data is consistent with reported data.[8]

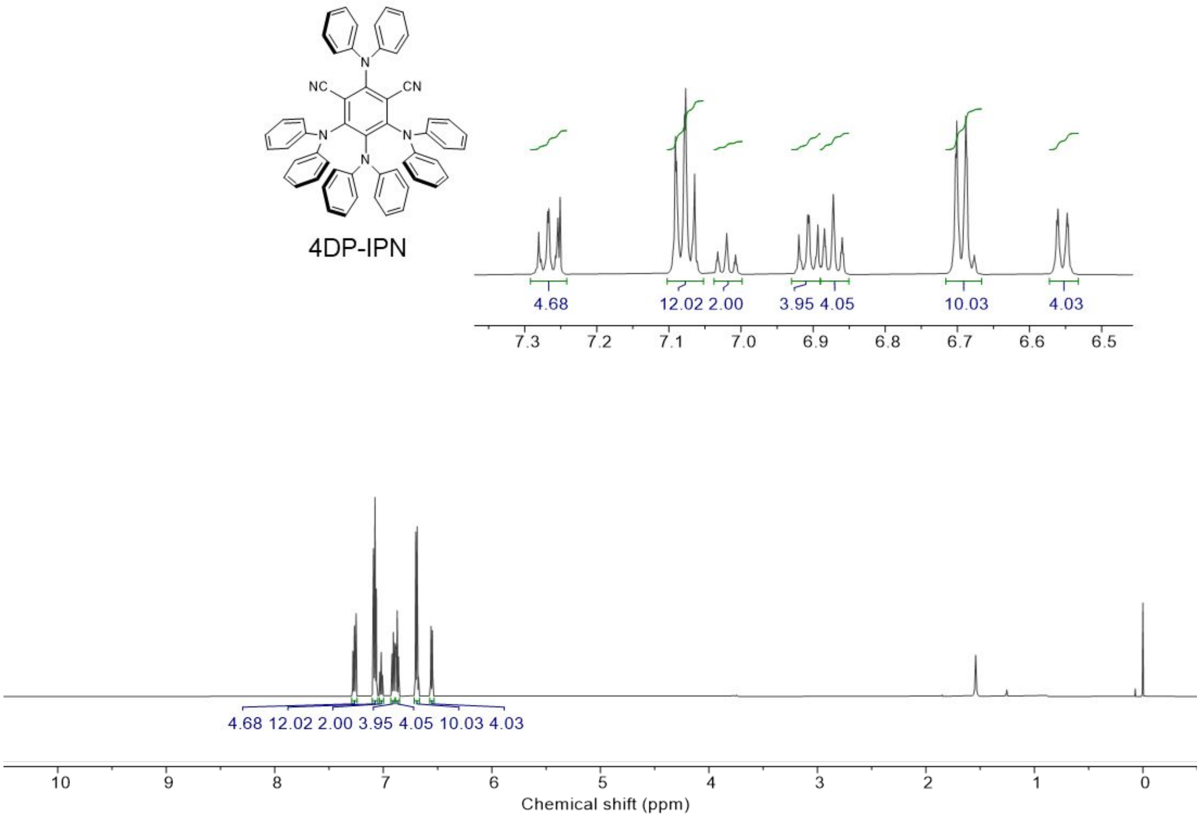

Figure S5 ^{1}H NMR data of 4DP-IPN at RT (600 MHz, $CDCl_3$).

S2 Steady State Measurements

S2.1 Experimental Details for Steady State Measurements

Steady state UV-visible absorption spectra were recorded using a PerkinElmer LAMBDA 950 UV-Vis spectrometer at the STFC Rutherford Appleton Laboratory. 2.5 mM solutions of 2Cz-BP, 2tCz-BP, 2PTZ-BP and 2PXZ-BP were prepared in DCM, and a 2.5 mM solution of 4DP-IPN was prepared in acetonitrile (MeCN). Samples were introduced into a stainless-steel Harrick cell (200 μm path length) fitted with 3 mm thick calcium fluoride (CaF_2) windows, and a single spectrum was recorded over the range 200 – 800 nm for each compound. Background spectra were recorded for pure solvent using the same Harrick cells as for sample acquisitions.

FTIR spectra were measured using a PerkinElmer Spectrum Two FTIR spectrometer at the University of Bristol. Samples of concentrations 1.0 mM (2Cz-BP), 1.25 mM (2tCz-BP), 1.5 mM (2PTZ-BP), 2.5 mM (2PXZ-BP) and 1.1 mM (4DP-IPN) in DCM were prepared as described above, and FTIR spectra were recorded over typical ranges of 1400 – 1800 cm^{-1}.

S2.2 Comparison of FTIR Experiments with DFT Calculations

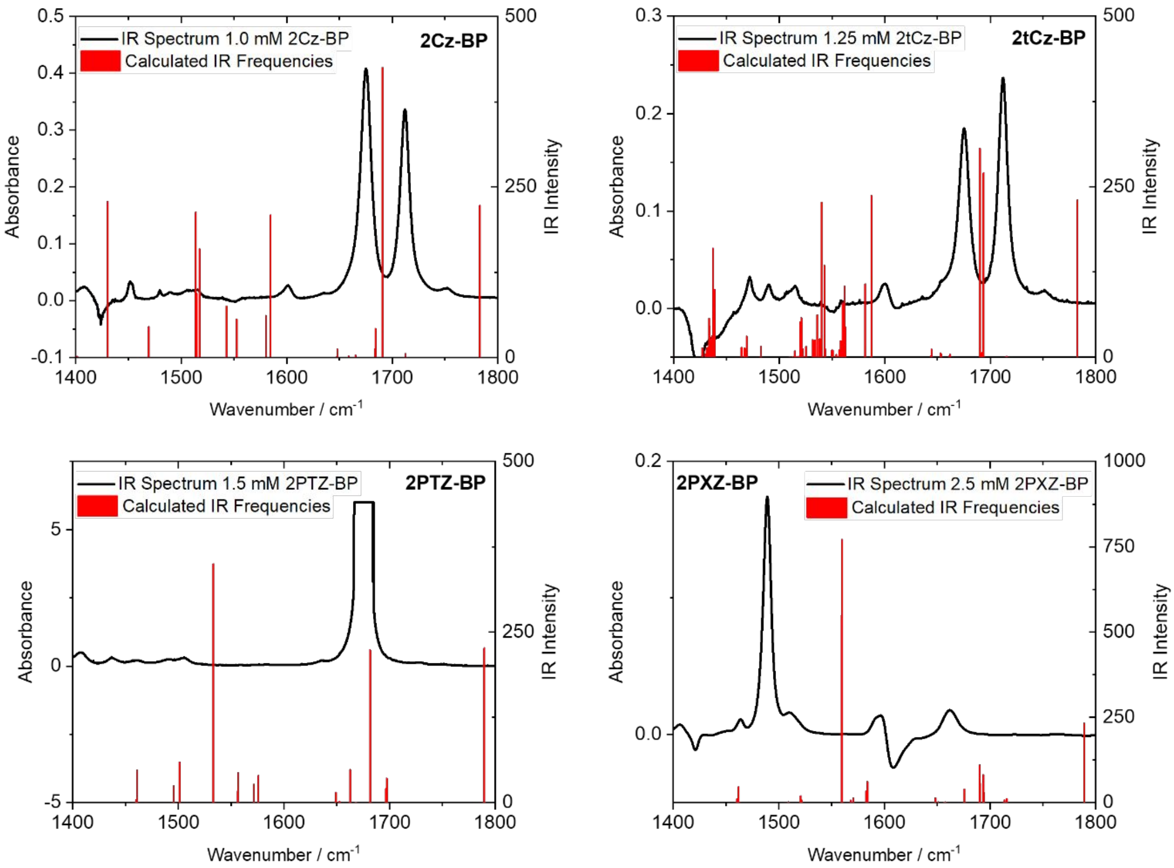

Figure S6 *FTIR spectra (black lines) for solutions of OPCs in DCM overlaid with vibrational frequencies (red bars) calculated at the ωB97XD/6-31+G(d) level of theory. Calculated IR frequencies are shown without any form of scaling applied.*

S2.3 Steady state photoluminescence emission spectroscopy

Photoluminescence (PL) emission spectra for 2Cz-BP, 2tCz-BP and 4DP-IPN measured in solvents of varying polarity are shown in Figure S7. These PL spectra demonstrate distinct solvatochromic shifts consistent with the assigned intramolecular charge-transfer (ICT) character of the electronically excited states. For 2Cz-BP in toluene the PL emission intensity is very low, so this spectrum has not been shown. The weak emission intensities observed for 2PTZ-BP and 2PXZ-BP are consistent with our assignment of these species as AIEgens. In DCM, DMF and THF, monomeric forms are favoured in solution and are only weakly emissive, so the recorded PL spectra are contaminated with Raman bands. However, because of their low solubility in toluene, aggregation causes appreciable emission. This aggregation induced emission typically shifts to longer wavelengths (500 – 700 nm, as shown in the PL spectra in THF and toluene plotted below). However, in DMF the emission bands assigned to aggregates of 2PTZ-BP and 2PXZ-BP exhibit smaller red shifts relative to the monomer emission bands compared to other solvents. Emission bands assigned to aggregates in DMF span wavelengths from (410 – 480 nm) and (410 – 460 nm) for 2PTZ-BP and 2PXZ-BP, respectively. TCSPC measurements of 2PTZ-BP and 2PXZ-BP in DCM and DMF recorded using a 570 nm detection wavelength were compared to measurements made with a 404 nm detection wavelength (Table 1 and Table S5). Time constants for the decay traces were consistent regardless of detection wavelength, verifying that fluorescence emission is originating from the same species (aggregates), and the shifted emission bands in 2PTZ-BP and 2PXZ-BP can be attributed to aggregation.

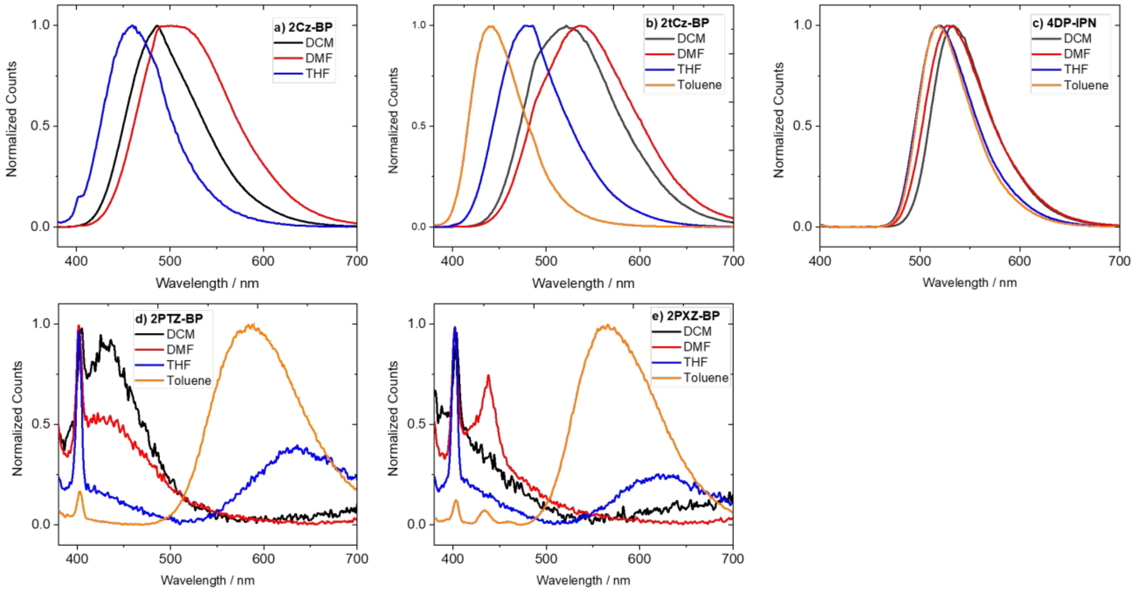

Figure S7 *Photoluminescence emission spectra for 10 µM solutions of (a) 2Cz-BP, (b) 2tCz-BP, (c) 4DP-IPN, (d) 2PTZ-BP and (e) 2PXZ-BP obtained using an excitation wavelength of 360 nm. Samples were dissolved in four solvents (DCM, DMF, THF and toluene), and were not sparged prior to irradiation. Sharper features evident in some spectra are Raman bands.*

S3 Natural Transition Orbital Diagrams for Key States

For carbazole-type OPCs, natural transition orbitals (NTOs) are only shown for $S_2 \leftarrow S_0$ locally excited (LE) electronic transitions. At the ωB97XD/6-31+G(d) level of theory, DFT fails to predict $S_1 \leftarrow S_0$ charge-transfer (CT) transitions observed at wavelengths greater than 350 nm, therefore the first calculated singlet state excitation (λ = 321 nm) is representative of the second electronic transition, not the first. Similarly, only the $S_2 \leftarrow S_0$ transition is shown for 2PTZ-BP due to the S_1 state being optically inaccessible. A full discussion is presented in the main text, section 3.1, together with NTO projections for 2PXZ-BP. DFT calculations also fail to predict the $S_1 \leftarrow S_0$ CT transition for 4DP-IPN arising between 475 and 500 nm, therefore only the $S_2 \leftarrow S_0$ CT transition is presented.

S3.1 Carbazoles

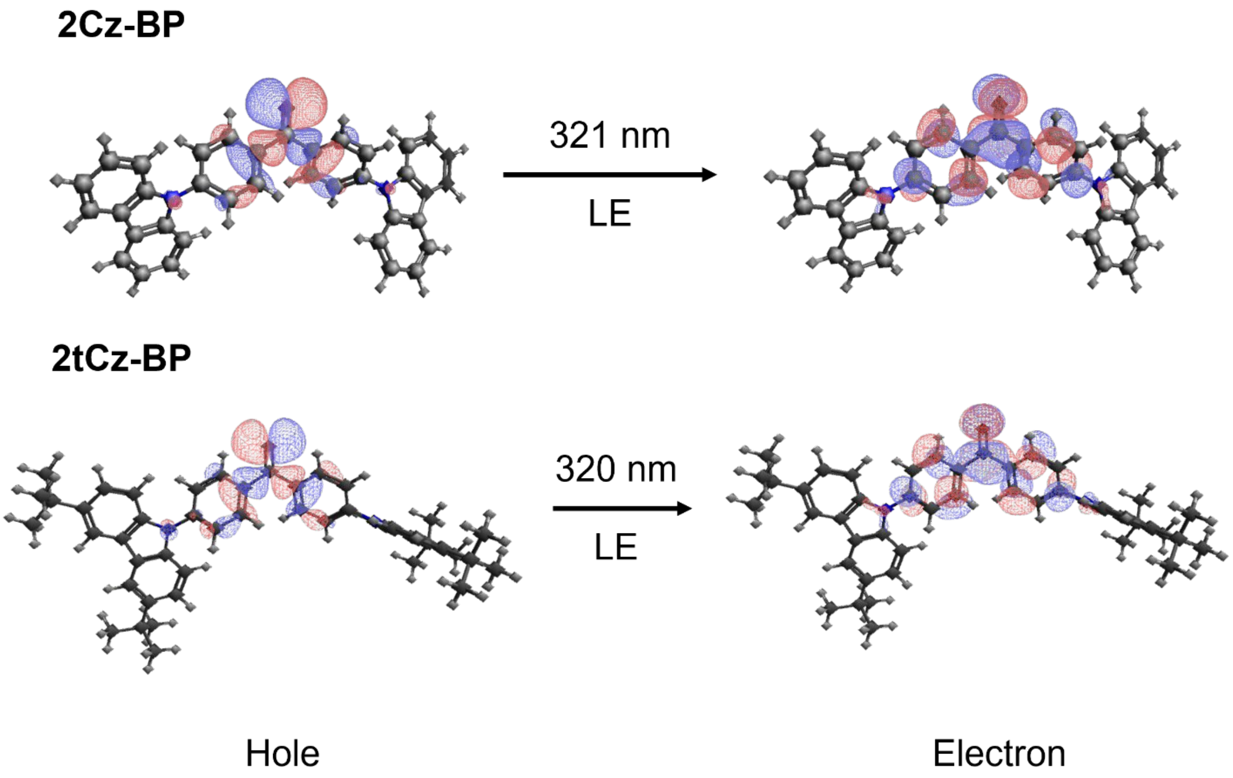

Figure S8 *NTOs involved in the $S_2 \leftarrow S_0$ LE electronic transition for carbazole-type OPCs 2Cz-BP (top) and 2tCz-BP (bottom).*

S3.2 2PTZ-BP

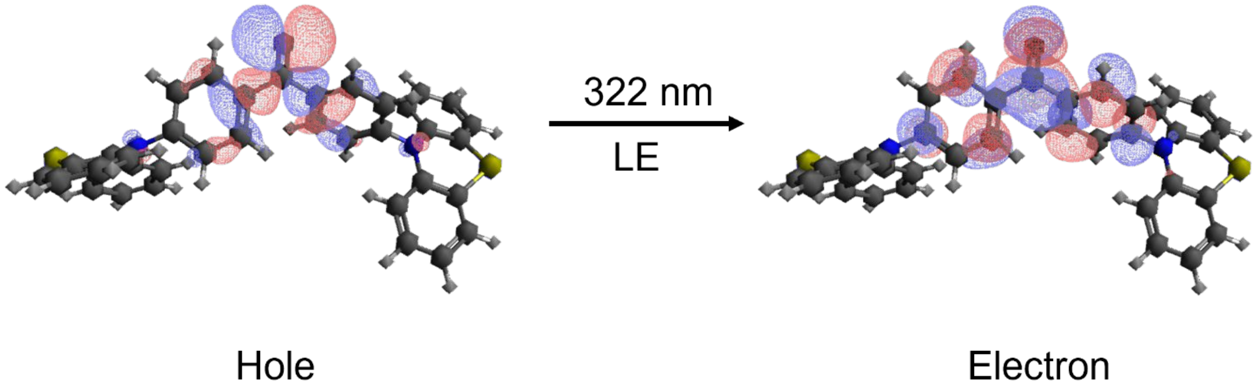

Figure S9 *NTOs involved in the $S_2 \leftarrow S_0$ LE electronic transition for 2PTZ-BP.*

S3.3 4DP-IPN

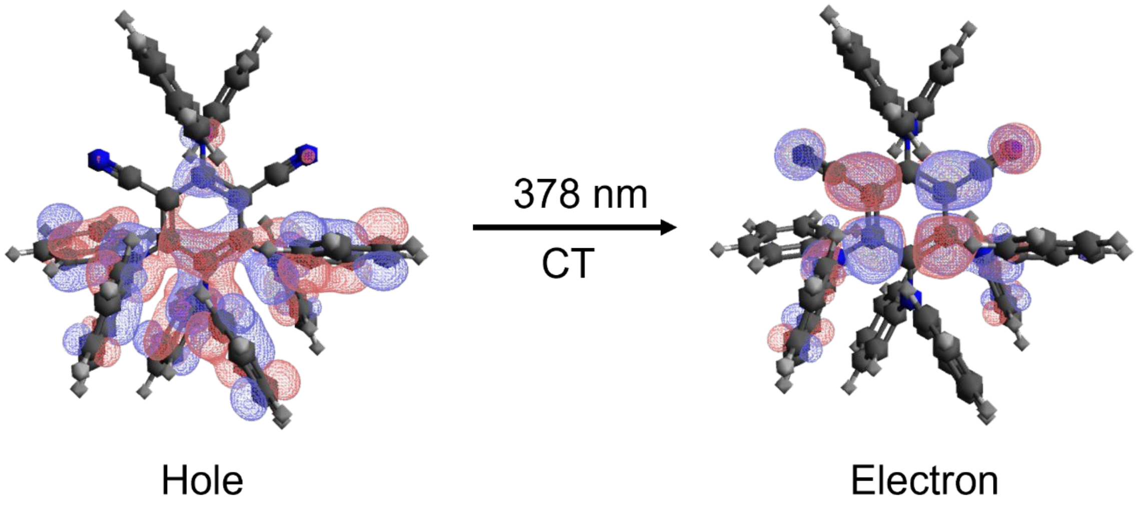

Figure S10 *NTOs involved in the $S_2 \leftarrow S_0$ CT electronic transition for 4DP-IPN.*

S4 Time-Resolved Fluorescence Traces

S4.1 Time-Resolved Fluorescence Traces for OPCs in DMF and DCM

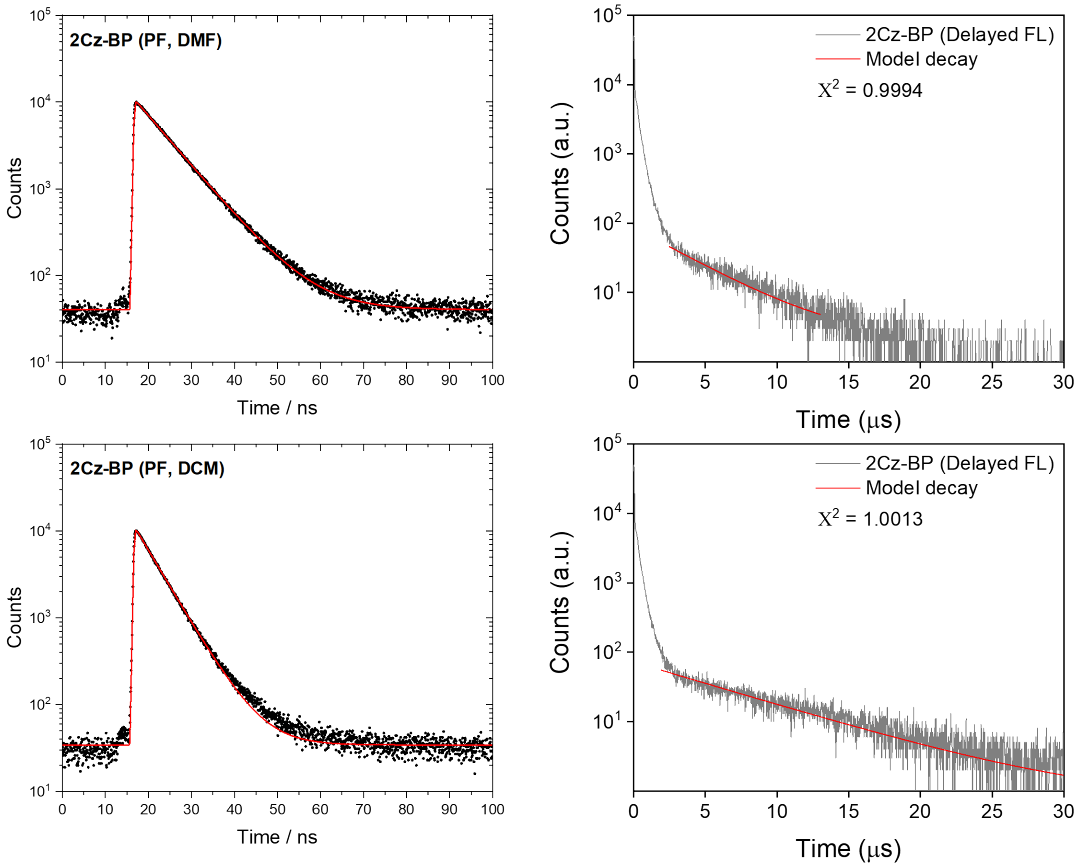

Figure S11 *Time-resolved fluorescence decay traces for 10 μM solutions of 2Cz-BP in DMF (top row) and DCM (bottom row). Solid red lines are exponential fits to raw data (black circles and grey lines). Decay traces were obtained using a 377 nm excitation pulse and a detection wavelength of 530 nm. Samples for prompt fluorescence traces (left column) were not sparged, whereas samples for delayed fluorescence traces (right column) were sparged with argon gas for 10 minutes prior to irradiation.*

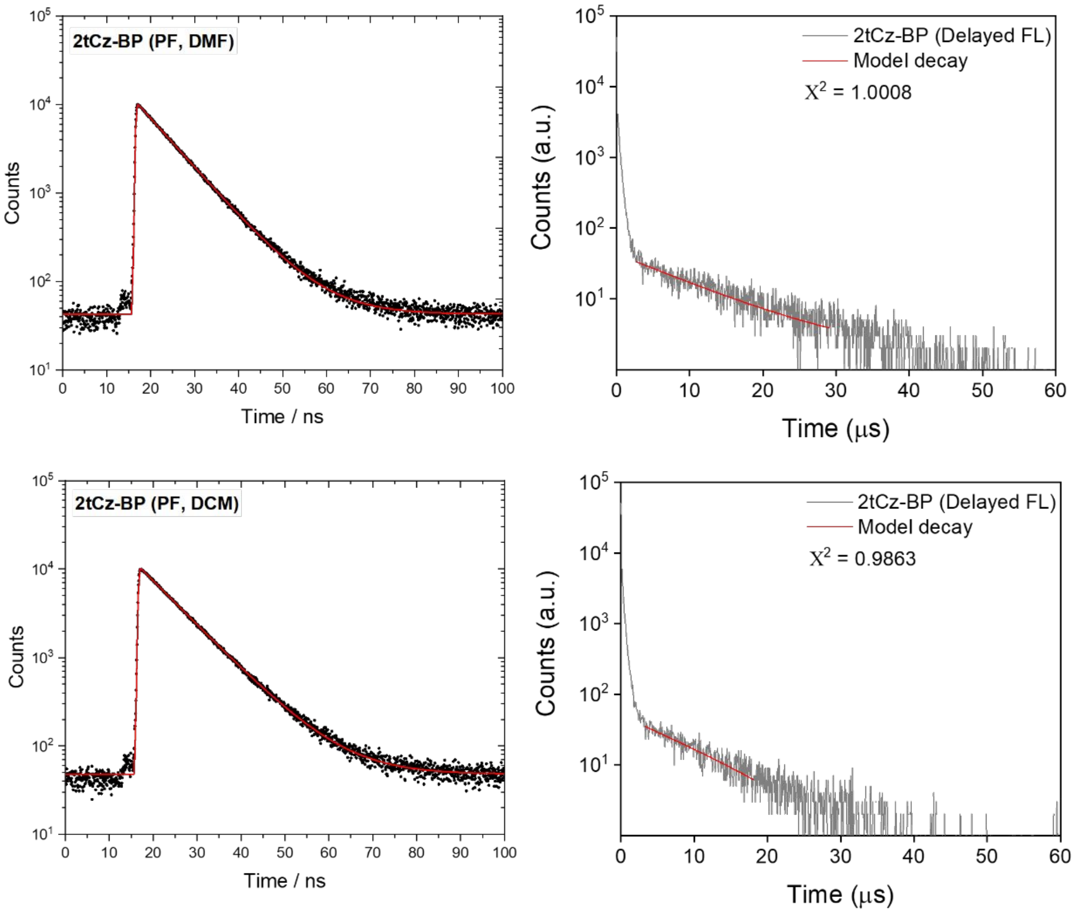

Figure S12 *Time-resolved fluorescence decay traces for 10 μM solutions of 2tCz-BP in DMF (top row) and DCM (bottom row). Solid red lines are exponential fits to raw data (black circles and grey lines). Decay traces were obtained using a 377 nm excitation pulse and a detection wavelength of 530 nm. Samples for prompt fluorescence traces (left column) were not sparged, whereas samples for delayed fluorescence traces (right column) were sparged with argon gas for 10 minutes prior to irradiation.*

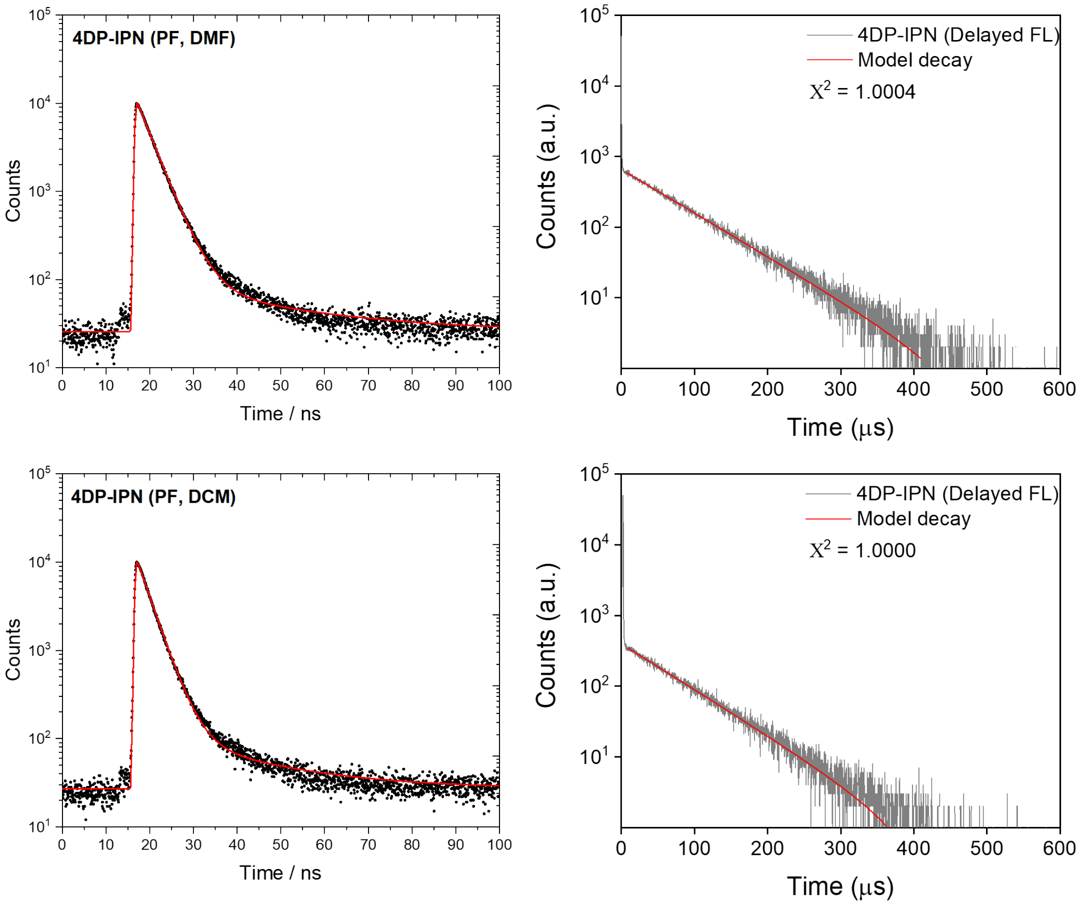

Figure S13 *Time-resolved fluorescence decay traces for 10 μM solutions of 4DP-IPN in DMF (top row) and DCM (bottom row). Solid red lines are exponential fits to raw data (black circles and grey lines). Decay traces were obtained using a 377 nm excitation pulse and a detection wavelength of 530 nm. Samples for prompt fluorescence traces (left column) were not sparged, whereas samples for delayed fluorescence traces (right column) were sparged with argon gas for 10 minutes prior to irradiation.*

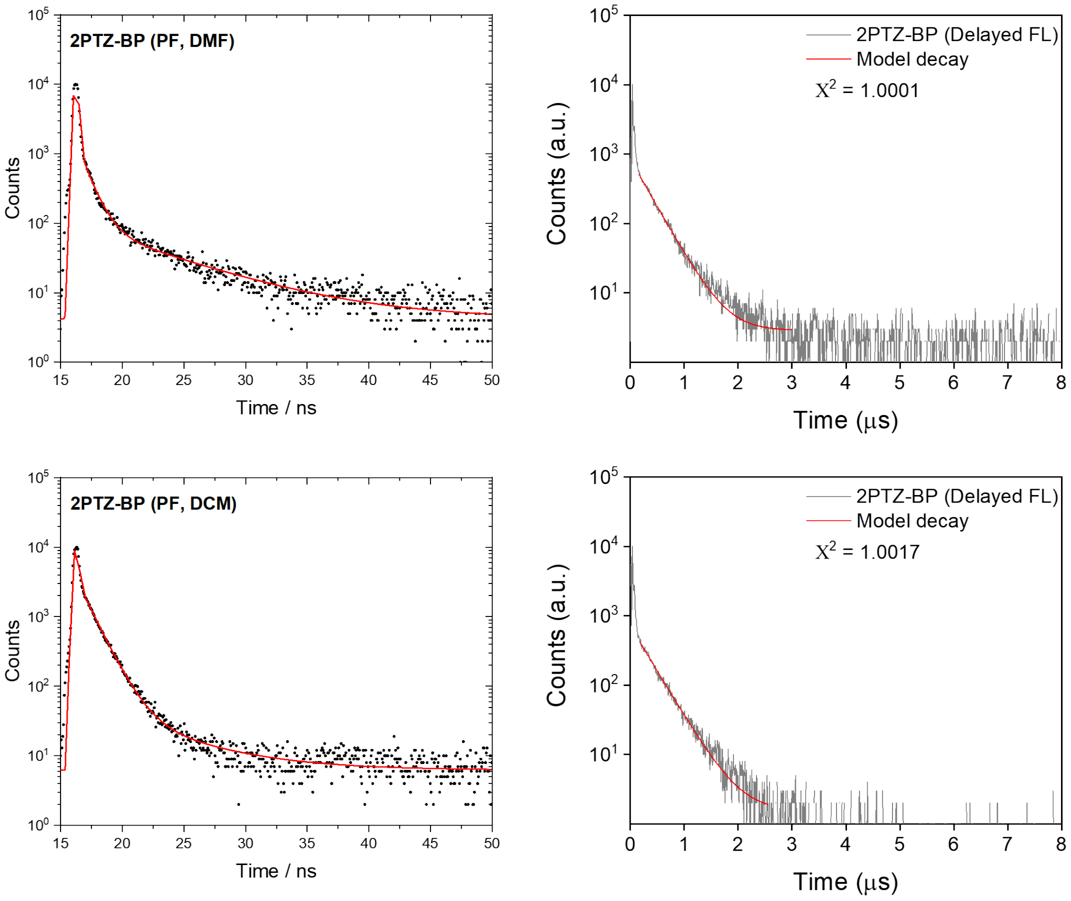

Figure S14 *Time-resolved fluorescence decay traces for 10 μM solutions of 2PTZ-BP in DMF (top row) and DCM (bottom row). Solid red lines are exponential fits to raw data (black circles and grey lines). Decay traces were obtained using a 377 nm excitation pulse and a detection wavelength of 404 nm. Samples for prompt fluorescence traces (left column) were not sparged, whereas samples for delayed fluorescence traces (right column) were sparged with argon gas for 10 minutes prior to irradiation.*

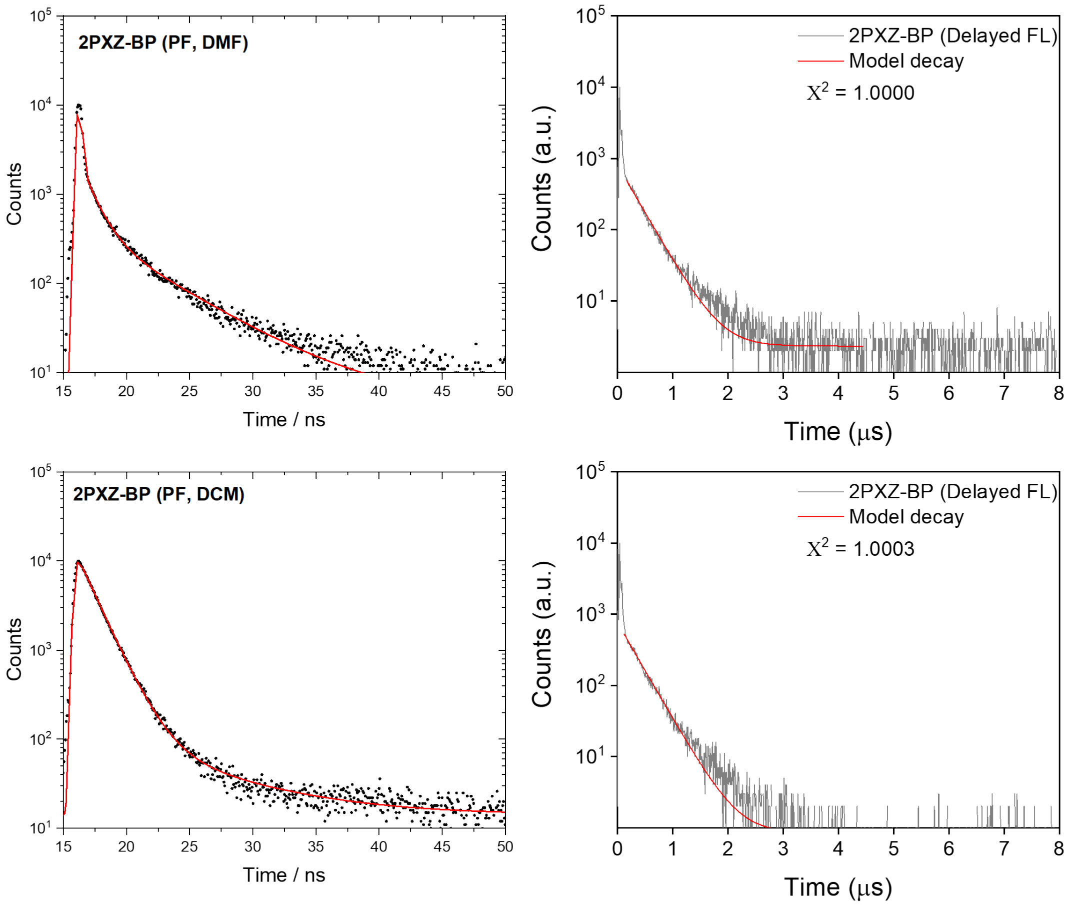

Figure S15 *Time-resolved fluorescence decay traces for 10 μM solutions of 2PXZ-BP in DMF (top row) and DCM (bottom row). Solid red lines are exponential fits to raw data (black circles and grey lines). Decay traces were obtained using a 377 nm excitation pulse and a detection wavelength of 404 nm. Samples for prompt fluorescence traces (left column) were not sparged, whereas samples for delayed fluorescence traces (right column) were sparged with argon gas for 10 minutes prior to irradiation.*

Table S1 Reproducibility test for delayed fluorescence lifetimes for OPCs in DMF measured using TCSPC

Measurement	2Cz-BP / µs	2tCz-BP / µs	2PTZ-BP / µs	2PXZ-BP / µs	4DP-IPN / µs
1	3.78	10.1	0.32	0.32	70.6
2	3.38	7.27	0.3	0.3	73.19
3	2.83	7.08	0.3	0.3	84.58
4	3.04	7.03	0.33	0.28	84.57
5	3.26	8.16	0.32	0.32	65.33
Mean	3.26	7.93	0.31	0.30	75.65
SD	0.36	1.30	0.01	0.02	8.62

Table S2 Reproducibility test for delayed fluorescence lifetimes for OPCs in DCM measured using TCSPC

Measurement	2Cz-BP / µs	2tCz-BP / µs	2PTZ-BP / µs	2PXZ-BP / µs	4DP-IPN / µs
1	6.95	9.58	0.34	0.32	67.24
2	6.44	17.72	0.34	0.32	61.87
3	7.45	15.01	0.35	0.31	60.31
4	4.14	10.76	0.36	0.32	53.18
5	6.12	9.94	0.35	0.33	67.13
Mean	6.22	12.60	0.35	0.32	61.95
SD	1.27	3.59	0.01	0.01	5.80

Table S3 Prompt fluorescence decay fit amplitudes for OPCs measured using TCSPC in DMF

Compound	Amplitude		
	A1	A2	A3
2Cz-BP	5490 ± 2	/	/
2tCz-BP	5379 ± 2	/	/
2PTZ-BP	45500 ± 9000	750 ± 30	46 ± 28
2PXZ-BP	33500 ± 3000	1050 ± 20	210 ± 16
4DP-IPN	5977 ± 3	41 ± 2	/

Table S4 Prompt fluorescence decay fit amplitudes for OPCs measured using TCSPC in DCM

Compound	Amplitude		
	A1	A2	A3
2Cz-BP	5766 ± 2	/	/
2tCz-BP	5435 ± 2	/	/
2PTZ-BP	44700 ± 7900	1800 ± 20	27 ± 15
2PXZ-BP	6543 ± 4	69 ± 5	/
4DP-IPN	6136 ± 4	52 ± 3	/

S4.2 Emission Wavelength Dependent TCSPC

To verify that fluorescence emission occurs from a single species, TCSPC experiments were performed for solutions of 2PTZ-BP and 2PXZ-BP in DCM and DMF. Fluorescence decay traces were recorded at three detection wavelengths (404 nm, 430 nm and 480 nm) for each sample, from which fluorescence lifetimes were determined. Detection wavelengths were selected to span most of the (very weak) fluorescence emission bands of 2PTZ-BP and 2PXZ-BP in these solvents. The fitted time constants are reported in Tables S5 – S8. As discussed in S2.3, time constants measured using a 570 nm detection wavelength are consistent with those measured using a 404 nm detection wavelength. This correspondence indicates that there is overlap between the emission bands of aggregates and monomers. These results show that the prompt and delayed fluorescence lifetimes are unchanged regardless of detection wavelength, and therefore support our interpretation that emission is occurring from a single species showing TADF behaviour.

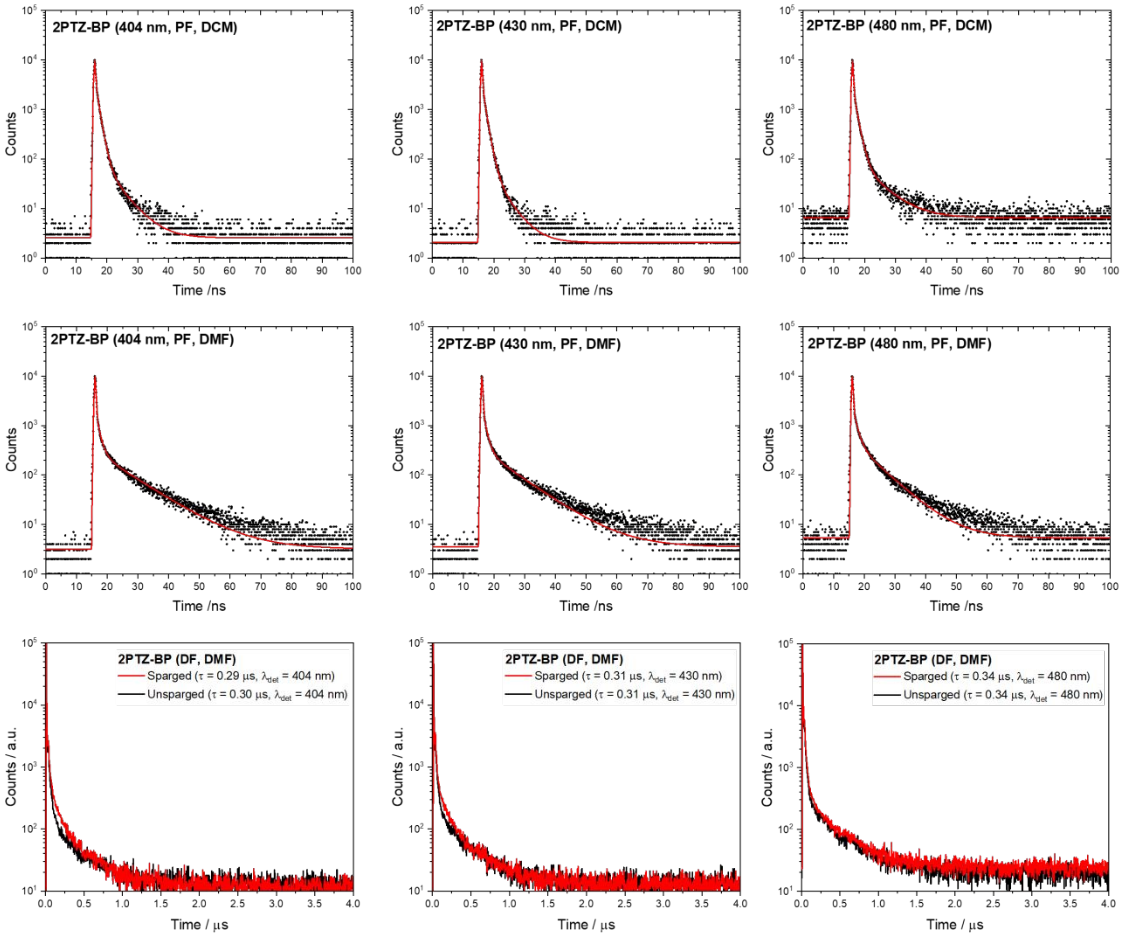

***Figure S16** Time-resolved fluorescence decay traces for 10 µM solutions of 2PTZ-BP in DCM (top row) and DMF (middle and bottom rows). Solid red lines are exponential fits to raw data (black circles, top and middle rows). For delayed fluorescence measurements (bottom row) data for a sparged (red, 10 mins sparging with Ar gas) and unsparged (black) samples are presented together. Decay traces were obtained using a 377 nm excitation pulse and detection wavelengths of 404 nm (left column), 430 nm (middle column) and 480 nm (right column). Samples for prompt fluorescence traces (top and middle rows) were not sparged.*

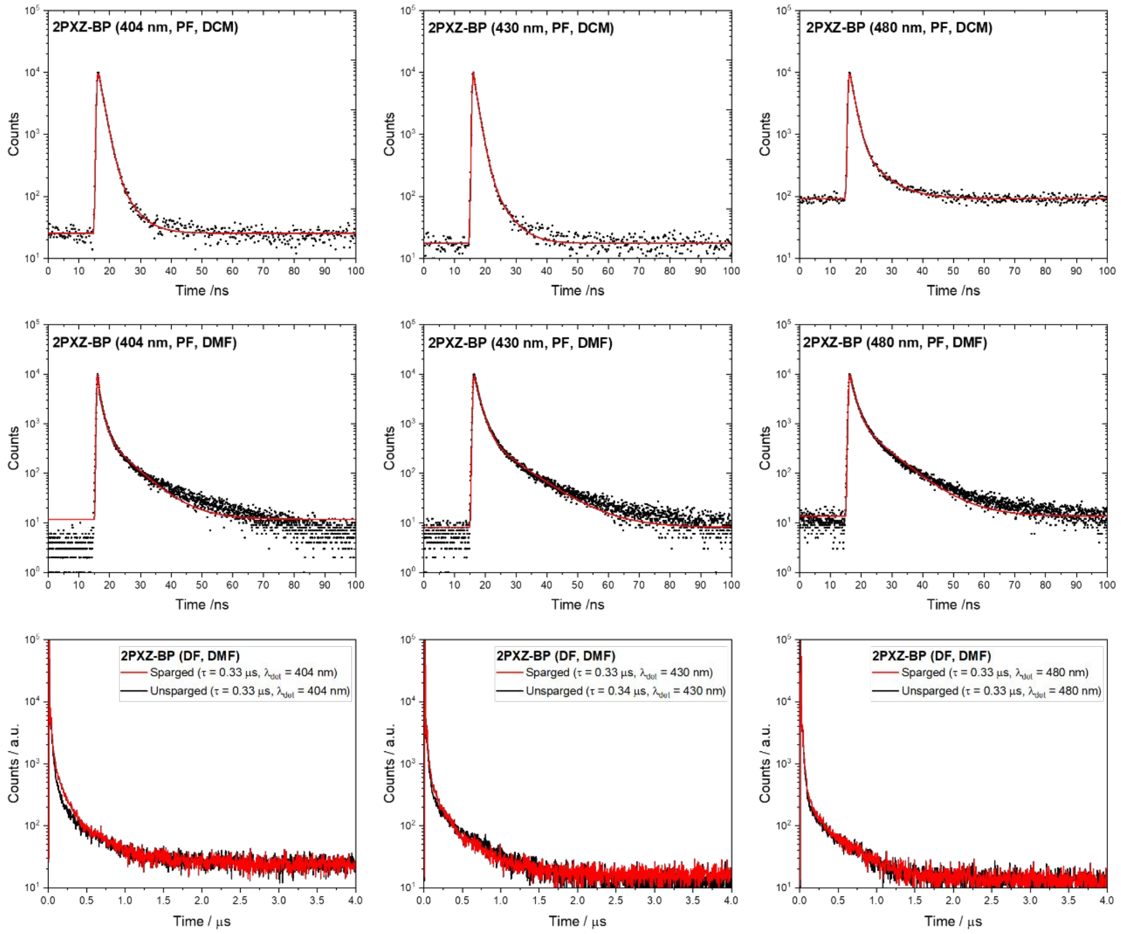

Figure S17 *Time-resolved fluorescence decay traces for 10 μM solutions of 2PXZ-BP in DCM (top row) and DMF (middle and bottom rows). Solid red lines are exponential fits to raw data (black circles, top and middle rows). For delayed fluorescence measurements (bottom row) data for a sparged (red, 10 mins sparging with Ar gas) and unsparged (black) samples are presented together. Decay traces were obtained using a 377 nm excitation pulse and detection wavelengths of 404 nm (left column), 430 nm (middle column) and 480 nm (right column). Samples for prompt fluorescence traces (top and middle rows) were not sparged.*

Table S5 Prompt fluorescence lifetimes for 2PTZ-BP and 2PXZ-BP in DCM and DMF obtained using TCSPC at three detection wavelengths

Compound	Time constant(s) / ns					
	DCM			DMF		
$\lambda_{Detection}$	404 nm	430 nm	480 nm	404 nm	430 nm	480 nm
2PTZ-BP	IRF and 1.07 ± 0.02	IRF and 1.09 ± 0.03	IRF and 1.21 ± 0.02	IRF and 1.16 ± 0.02	IRF and 1.09 ± 0.02	IRF and 1.21 ± 0.02
2PXZ-BP	1.51 ± 0.01	IRF and 1.36 ± 0.01	1.46 ± 0.01	IRF and 1.24 ± 0.02	IRF and 1.64 ± 0.00[#]	IRF and 1.55 ± 0.00[#]

**Time constants smaller than the instrument response function (IRF) are reported as IRF limited. [#]In these analyses, time constants were manually adjusted to reproduce experimental data, so are quoted without uncertainties.*

Table S6 Delayed fluorescence lifetimes for 2PTZ-BP and 2PXZ-BP in DMF obtained using TCSPC at three detection wavelengths

Compound	Time Constant / ns		
$\lambda_{Detection}$	404 nm	430 nm	480 nm
2PTZ-BP	290	310	340
2PXZ-BP	330	330	330

Table S7 Prompt fluorescence decay fit amplitudes for two OPCs measured using TCSPC in DCM at three detection wavelengths

Compound	Detection Wavelength / nm								
	404			430			480		
	A1	A2	A3	A1	A2	A3	A1	A2	A3
**2PTZ-BP**	28900 ± 2300	2290 ± 20	63 ± 12	20300 ± 680	1840 ± 30	52 ± 21	29850 ± 520	1530 ± 25	53 ± 10
**2PXZ-BP**	/	6880 ± 20	260 ± 25	19600 ± 7300	5460 ± 60	221 ± 21	/	6660 ± 20	374 ± 15

Table S8 Prompt fluorescence decay fit amplitudes for two OPCs measured using TCSPC in DMF at three detection wavelengths

Compound	Detection Wavelength / nm								
	404			430			480		
	A1	A2	A3	A1	A2	A3	A1	A2	A3
**2PTZ-BP**	54700 ± 1400	920 ± 16	147 ± 4	54700 ± 1600	1150 ± 15	165 ± 4	57100 ± 600	1120 ± 14	243 ± 8
**2PXZ-BP**	20900 ± 4300	3090 ± 30	337 ± 16	16010 ± 0#	4880 ± 0#	343 ± 1	16050 ± 0#	4780 ± 0#	650 ± 0#

#*In these analyses, amplitudes were manually adjusted to reproduce experimental data, so are quoted without uncertainties.*

S4.3 TCSPC for Aggregates of 2PTZ-BP and 2PXZ-BP

The observed photoluminescence (PL) emission intensities of 2PTZ-BP and 2PXZ-BP in neat DMF are much lower than for other OPCs studied. Therefore, to collect time-resolved fluorescence traces for these samples, the required energy of the excitation laser is between two and three times larger than that used for other OPCs. Acquisitions also require approximately ten times the photon counting of other samples, and the slit width of the detector is set to the maximum to enhance weak signals. On addition of water (90% by volume, FW=0.9) much greater PL emission intensity is observed from the samples. We attribute this enhanced luminescence to aggregation induced emission in this class of OPC. Furthermore, the delayed fluorescence lifetimes observed in neat DMF are approximately two to three times smaller than those in the mixed DMF / water solution, which is attributed to suppression of non-radiative decay pathways because of aggregation.

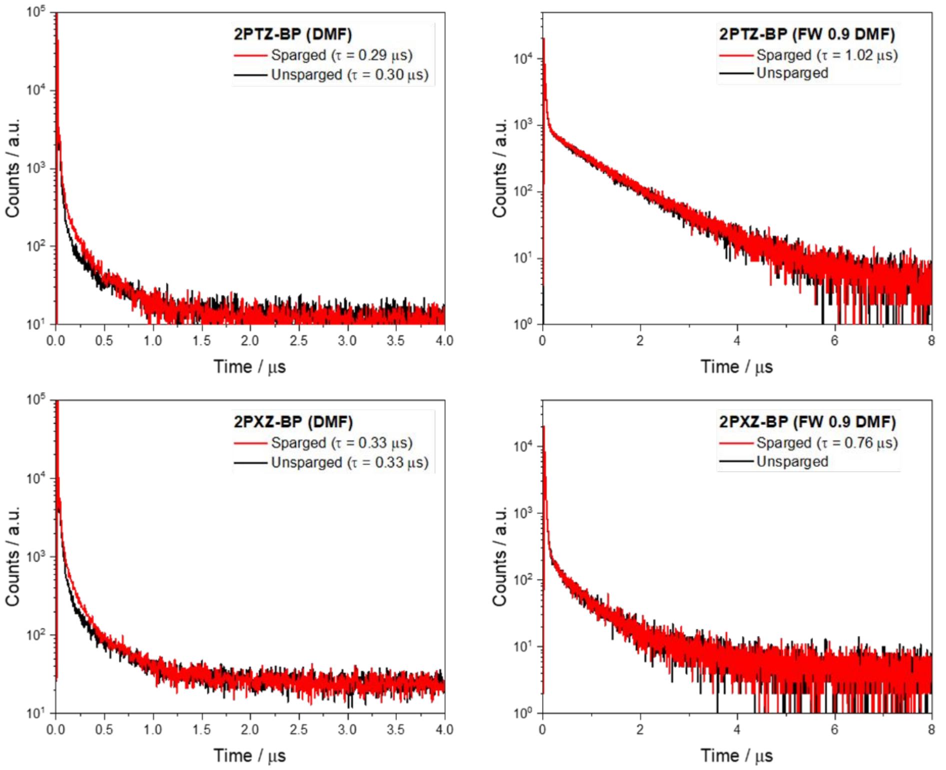

Figure S18 *Time-resolved fluorescence decay traces for 10 μM solutions of 2PTZ-BP (top row) and 2PXZ-BP (bottom row) in neat DMF (left column) and in DMF with 90 % by volume of water (right column; FW = fraction of water). Delayed component of PL traces were obtained using a 377 nm excitation pulse and detection wavelengths of 404 nm (neat DMF) and 560 nm (DMF / water mixture) because the emission shifts to longer wavelength. All samples for PL decay traces were sparged with argon gas for 10 minutes prior to irradiation.*

S5 Analysis of Transient Absorption Spectra

Transient electronic absorption spectra (TEAS) and transient vibrational absorption spectra (TVAS) were processed and analysed using the KOALA2 program.[9] For all measurements, a flat-shift baseline correction was applied to the data, and a spectrum recorded at negative time delay (i.e., with the probe pulse preceding the pump pulse) was subtracted from transient spectra obtained at all subsequent time delays. Correction to TEAS measurements to account for chirp introduced by the broadband white-light continuum (WLC) probe pulse was performed during analysis, as implemented in KOALA2. However, chirp correction was not necessary for TVAS measurements. Spectra were decomposed into constituent transient absorption bands using a combination of Gaussian functions and basis spectra as described below. Time constants were obtained by fitting exponential functions to the time-dependent integrated band intensities of the various components of the decomposed spectra. This fitting was performed in Origin software.

S5.1 Decomposition of TEA Spectra

TEAS analysis of 2PTZ-BP in DCM used a combination of two Gaussian functions to model the evolution of the S_2 excited state absorption (ESA), and a basis spectrum corresponding to the TEAS measurement at a 50-ps time delay to follow the S_1 ESA feature. Figure S19 shows a series of analysis frames at representative times points to highlight how the Gaussian functions and 50-ps basis spectrum interact to yield an overall fit that matches the experimental data well. Spectral decomposition was limited to the range 375 – 625 nm, therefore individual components of decomposition do not extend beyond this range, observed in figure S19 as a vertical cut-off in the total fit at longer wavelengths.

TEA spectra for carbazole-type OPCs (2Cz-BP and 2tCz-BP) were analysed by gating relevant transient features within a defined wavelength range, and then integrating across these regions at each time point to extract kinetics. Integration bands were selected to follow the hot S_1 ESA (approximately 400-425 nm), stimulated emission at early times assigned to vibrationally hot S_1 state (approximately 470-490 nm) and stimulated emission at late times assigned to the vibrationally cool S_1 state (approximately 495-515 nm).

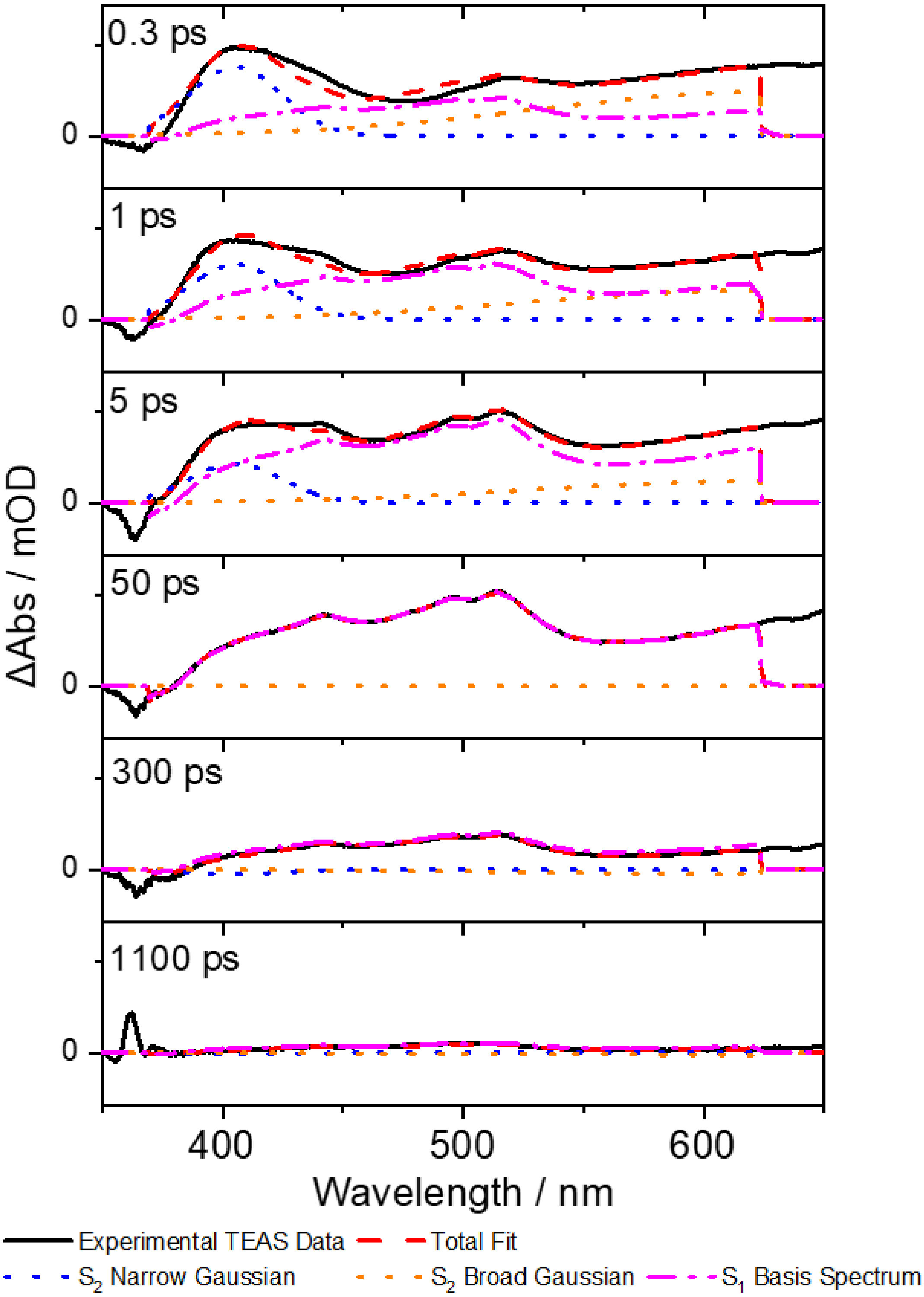

Figure S19 *Example decomposition of TEAS data at several time delays from 0.3 – 1100 ps for a solution of 2PTZ-BP in DCM photoexcited at 360 nm. Panels show the experimental TEAS data (black, solid line), the total fit (red, dashed line), Gaussian functions used to represent the S_2 state (blue and orange, dotted lines), and a basis spectrum acquired at 50 ps to represent the S_1 state ESA (pink, dash and dot line).*

S5.2 Decomposition of TVA Spectra Measured in DCM

For TVAS measurements acquired using the LIFEtime facility[2, 3] (section S1.1), the two ~ 200 cm^{-1} bandwidth IR probe pulses were dispersed and detected, calibrated and analysed independently. Where there is overlap of the probe regions observed by the left-hand and right-hand detectors (lhd and rhd) monitoring the two probe pulses, transient features were analysed only once using the detector for which the feature is better resolved.

Analysis frames at representative time points for photoexcited 2Cz-BP and 2PXZ-BP in DCM are shown in figures S20 and S21, respectively. Early time basis spectra (5 ps and 1 ps) were used to model bands corresponding to the S_1 electronic states of 2Cz-BP and 2PXZ-BP and have been modified such that any negative ΔA values are instead set to zero. The purpose of this modification was to avoid mutual dependency between the S_1 ESA features and the GSB features, which were fitted using Gaussian functions. For carbazole-type species, ESAs assigned to T_1 states were also observed and were fitted using Gaussian functions.

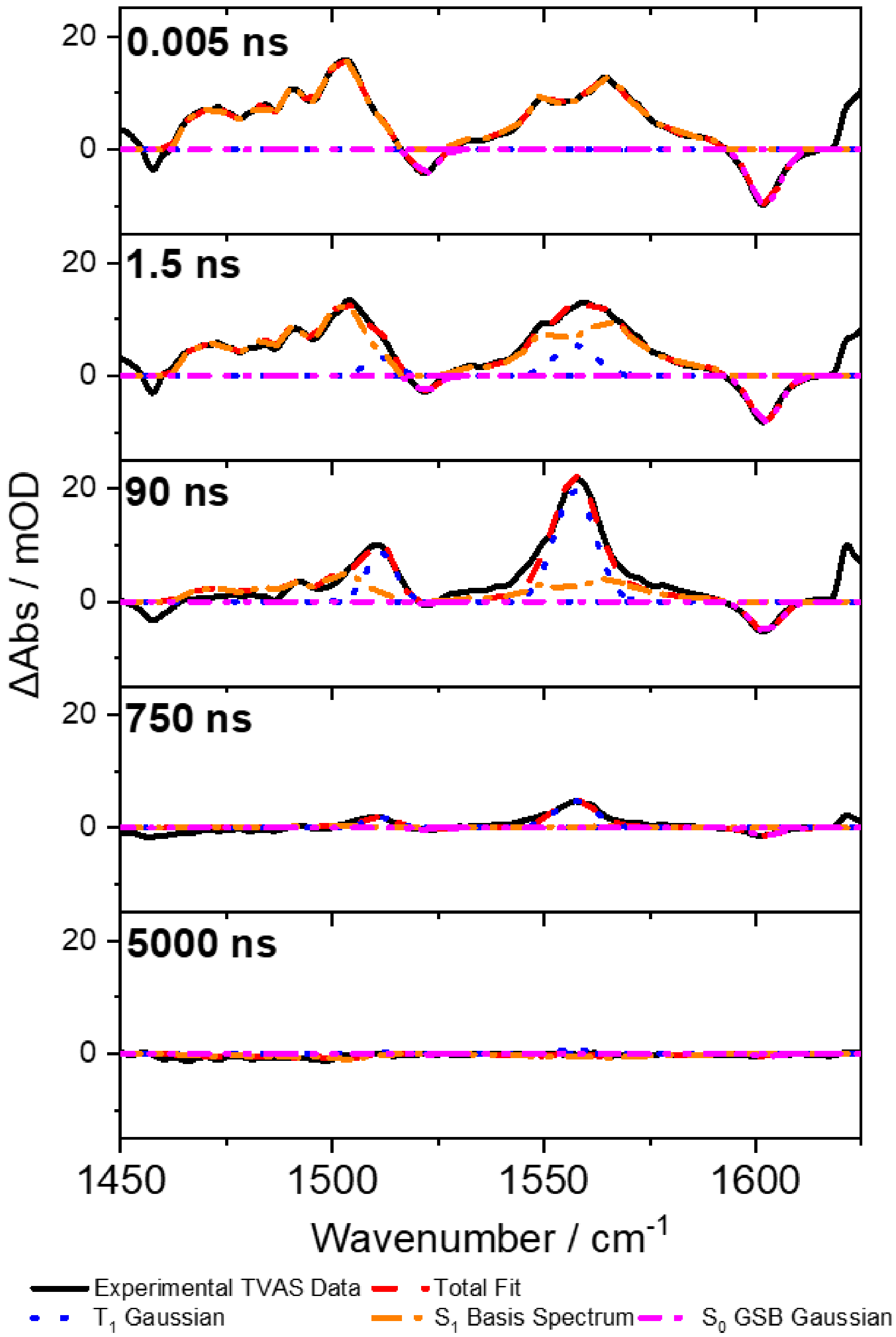

Figure S20 *Example decomposition of TVAS data at several time delays from 0.005 – 5000 ns for a solution of 2Cz-BP in DCM photoexcited at 360 nm. Panels show the experimental TVAS data (black, solid line), the total fit (red, dashed line), Gaussian functions used to represent the T_1 state bands (blue, dotted line) and S_0 GSB features (pink, dash-dot-dot line), and a basis spectrum acquired at 5 ps to represent the S_1 state (orange, dash-dot line).*

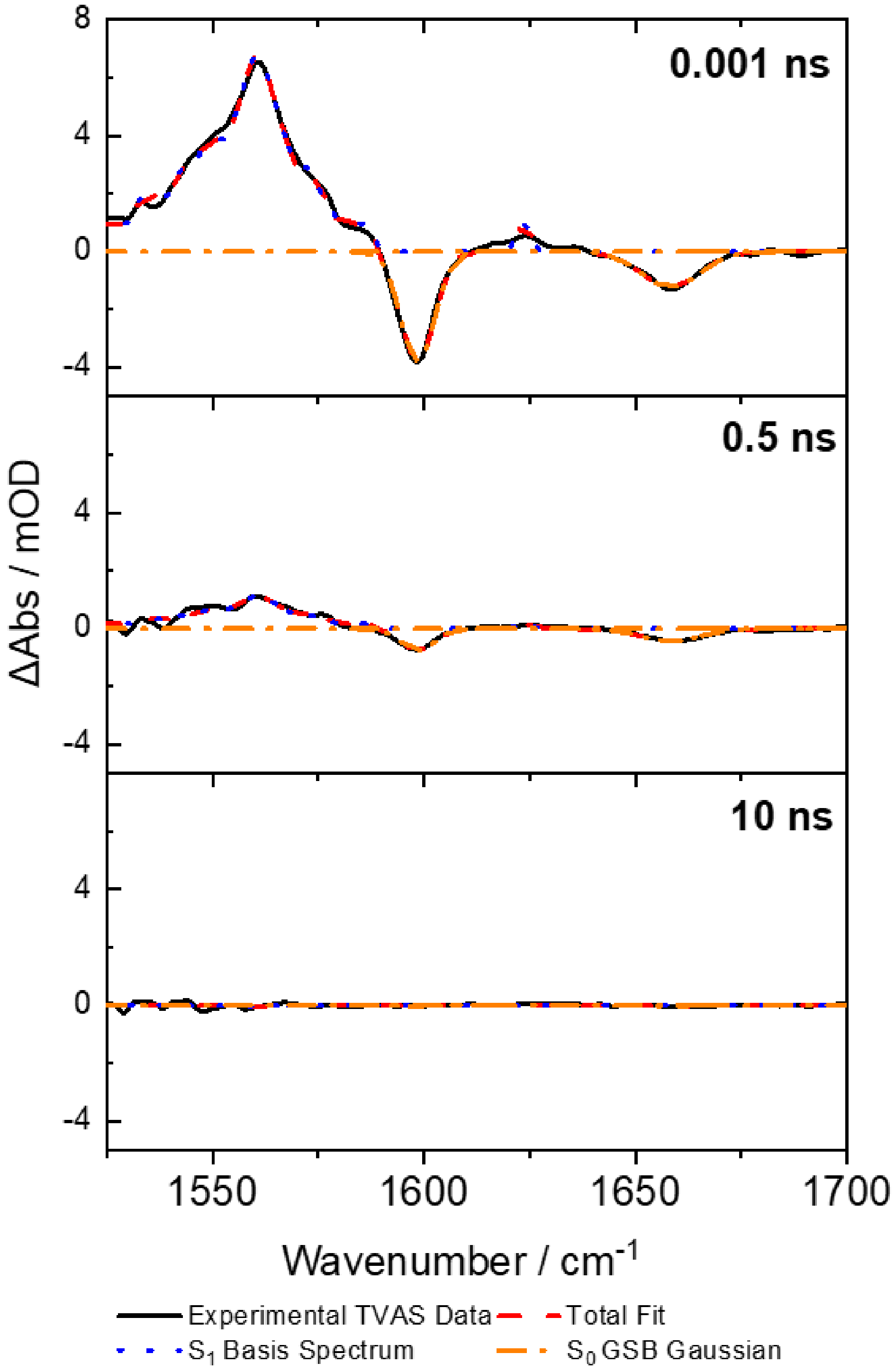

Figure S21 *Example decomposition of TVAS data at several time delays from 0.001 – 10 ns for a solution of 2PXZ-BP in DCM photoexcited at 425 nm. Panels show the experimental TVAS data (black, solid line), the total fit (red, dashed line), Gaussian functions to represent the S_0 GSB features (orange, dash-dot line) and a basis spectrum acquired at 1 ps to represent the S_1 state ESA (blue, dot line).*

S5.3 Additional TVAS and Kinetics Measurements in DMF

As discussed in the main text and Section S1, TVA spectra for all five OPCs in DMF were recorded over the range 1400-1700 cm^{-1} to observe ESA bands attributed to aromatic ring modes, as well as GSB features representative of the electronic ground state. DMF exhibits strong absorption bands in this IR region, therefore the spectra presented in figure S22 are restricted to the 1530-1610 cm^{-1} range.

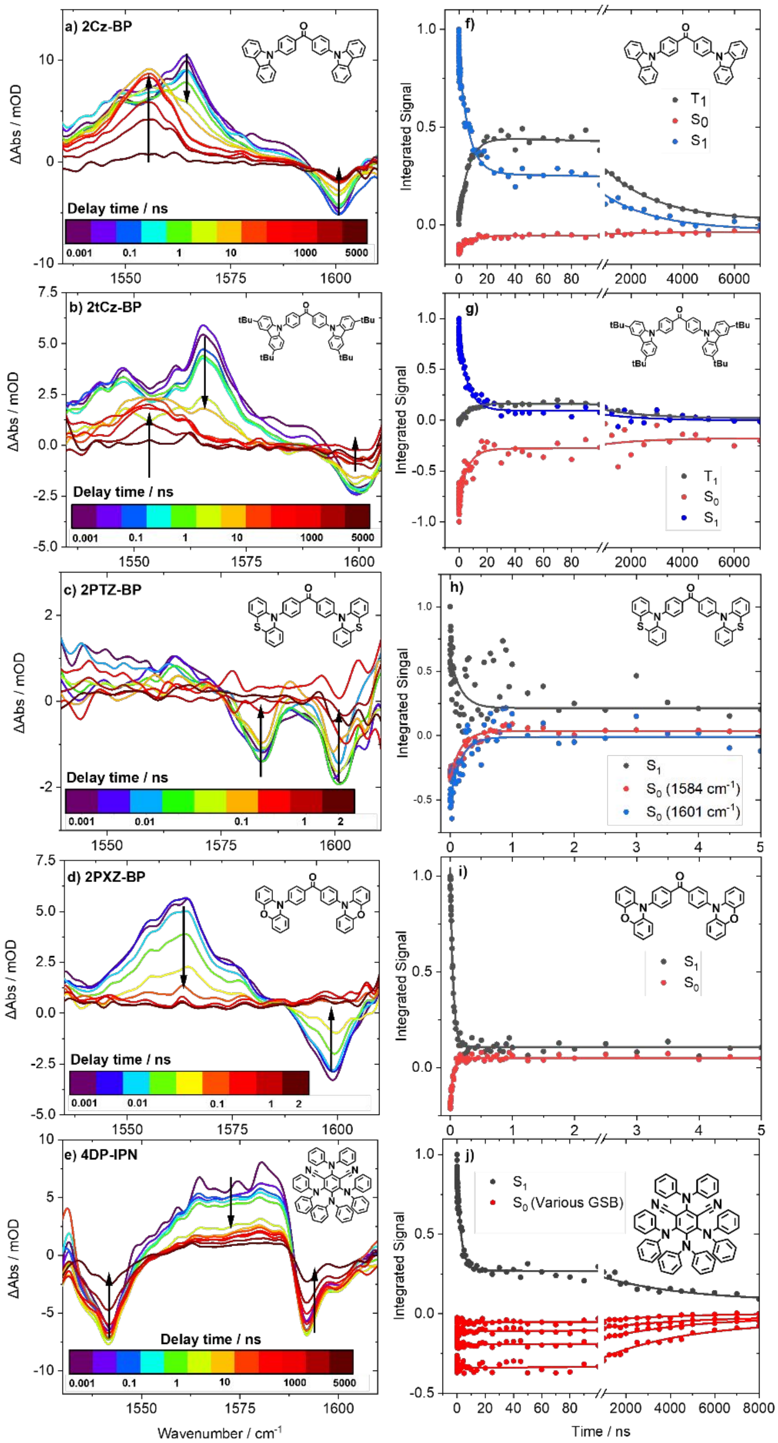

Figure S22*Transient vibrational absorption spectra obtained for time delays from 1 ps to 5 µs for solutions of (a) 2Cz-BP, (b) 2tCz-BP, (c) 2PTZ-BP, (d) 2PXZ-BP and (e) 4DP-IPN in N,N-dimethyl formamide. Spectra were obtained using a 360 nm UV pump pulse for 2Cz-BP, 2tCz-*

BP and 2PTZ-BP (a-c), and a 425 nm pump pulse for 2PXZ-BP and 4DP-IPN (d-e). Spectra are coloured to indicate the delay time of the broadband IR probe pulse, and black arrows show the directions of changes of band intensity with time. (f-i) Kinetic traces for the photocatalysts obtained from TVAS measurements in DMF. Solid lines are global exponential fits to data points (closed circles). Fitted exponential time constants are presented in the main text (table 2).

TVAS data for carbazole-type OPCs show that the photophysics of this class of molecule are consistent between DCM and DMF solvents. In both solvents, the kinetics of 2Cz-BP and 2tCz-BP are well modelled using bi-exponential decay functions with a prompt lifetime (τ_P, section 3.3 in the main text) and a delayed lifetime (τ_D) of 6 – 7 ns and 1 – 2 μs, respectively, in DMF. These lifetimes are comparable to the τ_P = 4 – 8 ns and τ_D = 0.5 – 2.5 μs observed in DCM. TVAS in DMF also shows direct evidence of triplet formation concurrent with S_1 state decay and partial GSB recovery on the timescale of the prompt lifetime components. Therefore, we assign the carbazole-type OPCs as TADF emitters in DMF using the same arguments as for these compounds in DCM in the main text.

Kinetics extracted from TVA spectra for 4DP-IPN in DMF also closely resemble those extracted in DCM, with τ_P ~ 3 ns in both solvents, and τ_D ~ 4 μs and 2.7 μs in DMF and DCM respectively. Decomposition of the TVAS data for 4DP-IPN is presented in section S5.4

TVAS experiments for 2PTZ-BP and 2PXZ-BP in DMF reveal kinetics similar to those in DCM. In both solvents we observe rapid mono-exponential decay of GSB features and ESA bands (assigned to the S_1 state) on sub-nanosecond timescales. Kinetic analysis of this class of OPC yields prompt lifetimes in DMF of 170 ps and 46 ps for 2PTZ-BP and 2PXZ-BP, which are shorter than the prompt lifetimes of 294 ps and 350 ps for 2PTZ-BP and 2PXZ-BP observed in DCM.

S5.4 Decomposition of TVA Spectra Measured in DMF

Decomposition of TVA spectra for 2Cz-BP, 2tCz-BP and 2PTZ-BP in DMF was approached in the same way as decomposition of analogous spectra in DCM (S5.2). For carbazole-type OPCs a modified early-time basis spectrum was used to represent the S_1 ESA features, and Gaussian functions were used to model the GSB features and emergent T_1 ESA. For 2PTZ-BP in DMF, the GSB features were each modelled with a Gaussian function (distinguished in figure S22, panel h, by their central wavenumber), and the evolution of the S_1 ESA was followed with a modified basis spectrum.

Figures S23 and S24 show the decompositions for 2PXZ-BP and 4DP-IPN respectively as these differ from methods used to decompose TVAS data in DCM solvent. Due to the restricted analysis region in DFM, 2PXZ-BP spectra were simply decomposed using two Gaussian functions representing the S_1 and S_0 state absorptions. 4DP-IPN spectra were instead decomposed using a modified early-time basis spectrum (2 ps) for the ESA band, and four Gaussian functions for the GSB features. Because each fitted Gaussian function evolved with the same time constants (on account of each function modelling recovery of the S_0 electronic state population), the kinetics for each function are presented in the same colour in figure S22 panel j for clarity.

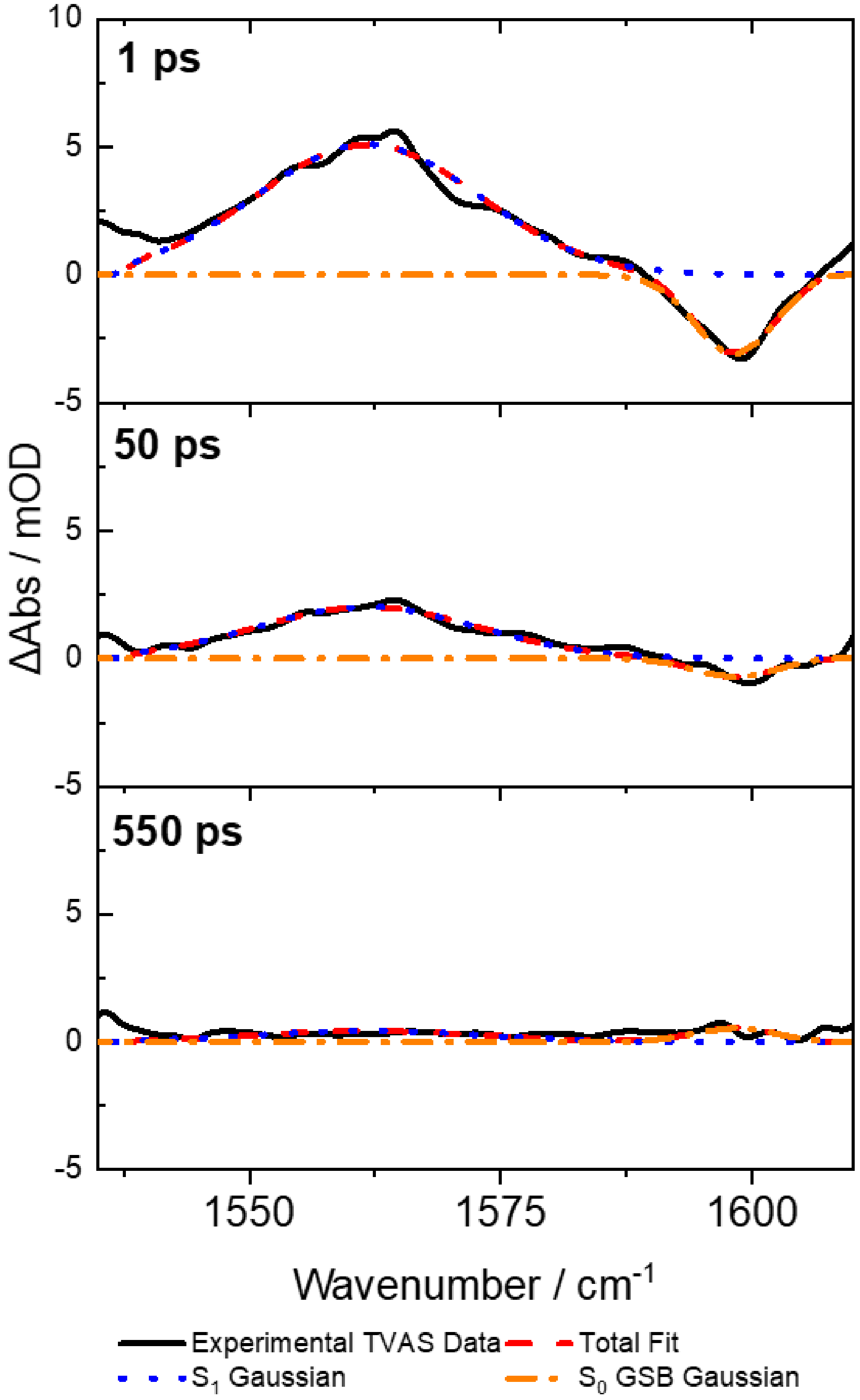

Figure S23 *Example decomposition of TVAS data at several time delays from 1 – 550 ps for a solution of 2PXZ-BP in DMF excited at 425 nm. Panels show the experimental TVAS data (black, solid line), the total fit (red, dashed line), and Gaussian functions used to represent the S_1 ESA feature (blue, dot line) and S_0 GSB feature (orange, dash-dot line).*

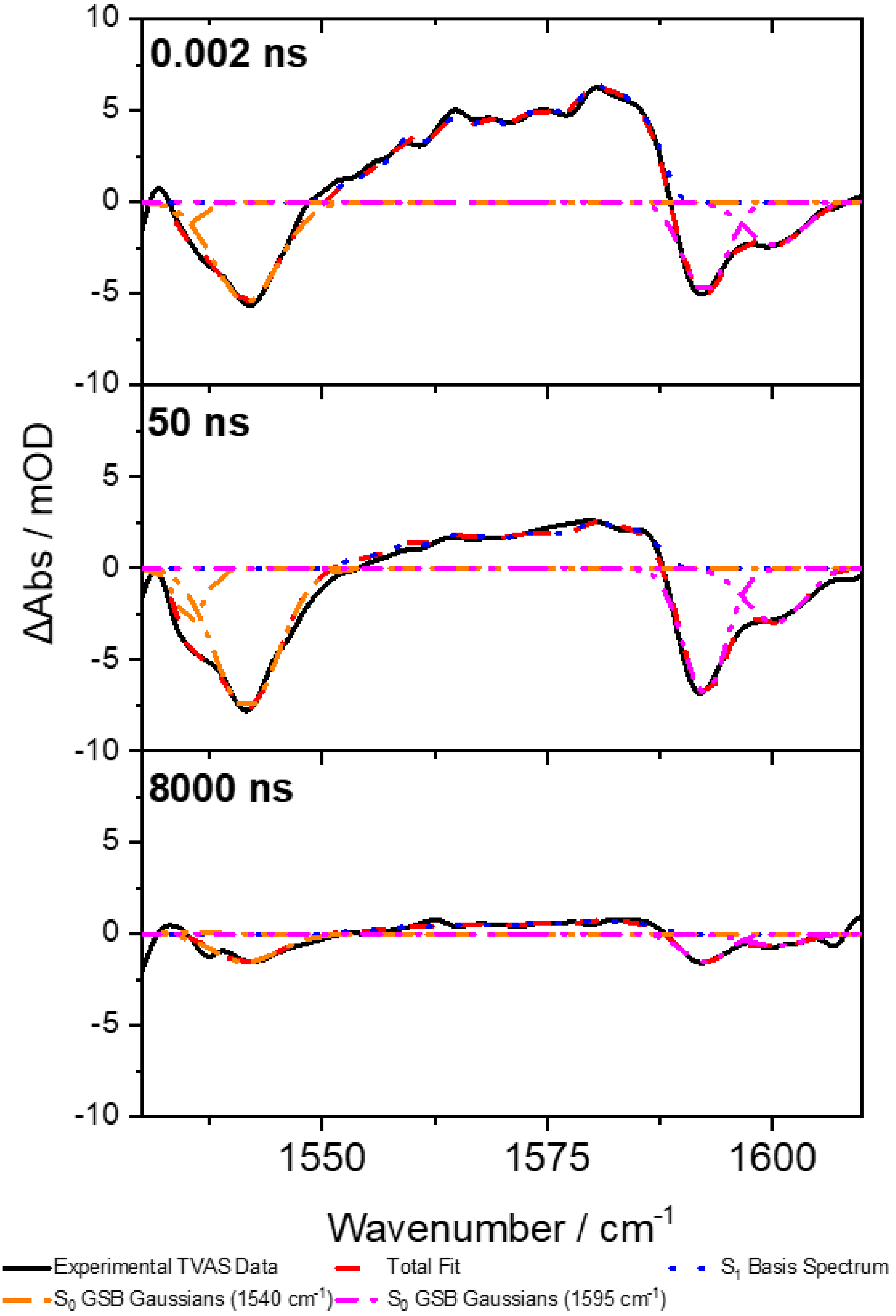

Figure S24 *Example decomposition of TVAS data at several time delays from 0.002 – 8000 ns for a solution of 4DP-IPN in DMF excited at 425 nm. Panels show the experimental TVAS data (black, solid line), the total fit (red, dashed line), a basis spectrum acquired at 2 ps used to represent the S_1 state (blue, dot line) and four Gaussian functions used to represent GSB features centred around 1540 cm^{-1} (orange, dash-dot line) and 1595 cm^{-1} (pink, dash-dot-dot line).*

S5.5 Triplet Quantum Yields

As discussed in the main text, quantum yields of triplet formation for the OPCs can be estimated from TVA spectra by comparing the amplitudes of the prompt and delayed components of GSB recovery kinetics.

$$\Phi(T_1) = \frac{A_D}{A_p + A_D} \qquad \textbf{(Equation S1)}$$

Where $\Phi(T_1)$ is the quantum yield for T_1 formation, A_P is the amplitude of the prompt GSB recovery component, and A_D is the amplitude of the delayed GSB recovery component.

Table S9 Estimated triplet (T_1) quantum yields for the five studied photocatalysts in dichloromethane and *N,N*-dimethyl formamide obtained by transient vibrational absorption spectroscopy.

Compound	Triplet quantum yield	
	DCM	DMF
2Cz-BP	0.62 $\pm$ 0.16	0.21 $\pm$ 0.15
2tCz-BP	0.33 $\pm$ 0.11	0.17 $\pm$ 0.05
2PTZ-BP*	0.0#	(7.2 $\pm$ 2.2) $\times$ 10^{-4}
2PXZ-BP*	0.044 $\pm$ 0.005	0.0011 $\pm$ 0.0003
4DP-IPN	0.83 $\pm$ 0.02	0.99 $\pm$ 0.13

*Note that because estimates have been made using TVAS data, the values quoted for 2PTZ-BP and 2PXZ-BP are representative of the non-aggregated states.

#No evidence for triplet formation was observed in this measurement.

S6 Additional Time Constants

Table S10 Ultrafast time constants τ_{UF} for 2Cz-BP, 2tCz-BP and 2PTZ-BP in DCM determined via TEAS (section 3.3, main text).

OPC	τ_{UF} / ps
2Cz-BP	1.7 ± 0.6
2tCz-BP	3.1 ± 0.2
2PTZ-BP	4.7 ± 0.2

For carbazole-type OPCs τ_{UF} describes vibrational cooling by energy transfer to solvent on the order of 1 - 3 ps. For 2PTZ-BP, τ_{UF} instead represents the ultrafast S_2 – S_1 IC lifetime.

Table S11 Reference time constants for 4Cz-IPN

Solvent	τ_{PF} / ns	τ_{DF} / μs
Acetonitrile	18.7	1.39
DCM	24.6	2.04
Toluene	11.3	4.6

Summary of prompt (τ_{PF}) and delayed (τ_{DF}) fluorescence time constants for 4Cz-IPN in acetonitrile,[10, 11] dichloromethane,[10] and toluene[12] sourced from previous literature. Time constants for 4Cz-IPN broadly agree with those derived for the molecular analogue 4DP-IPN in this study, showing a prompt component on the order of a few nanoseconds, and a longer delayed component on the microsecond timescale.

S7 O-ATRP performance for the organic photocatalysts

Table S12 summarizes ground and excited-state redox potentials for the five studied OPCs, their photochemical parameters, and performance as O-ATRP catalysts for the following polymerization reaction.

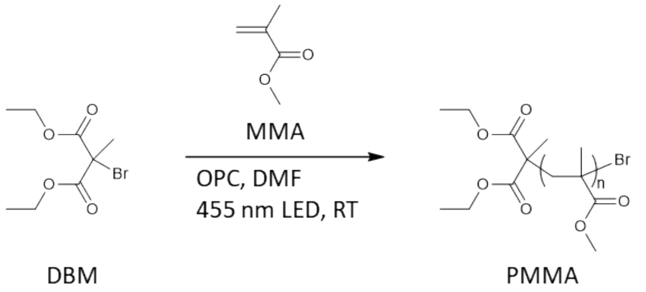

The scheme shows polymerization of MMA using OPCs with DBM as the initiator. Polymerizations were carried out in inert environments (N_2) at room temperature using [Monomer]:[Initiator]:[OPC] = 200:1:0.02 in DMF as a solvent (Monomer/solvent : 1/1 (v/v)).

In table 12, the polymerization parameters are defined as follows: α is the polymerization yield; Đ is the polymer dispersity; *I** is the initiator efficiency, quantifying activation of the initiator by electron transfer. *I** is defined as $I^* = M_{n,calc.}/M_{n,exp}$ where M_n is the number average molecular weight, 'calc.' stands for 'calculated' and 'exp.' stands for 'experimental'. The 'calculated' values are theoretical values from the yield (or conversion) under the assumption that all initiators are activated. The 'experimental' values are obtained by gel permeation chromatography. For example, if $M_{n,calc}$ is small and $M_{n,exp}$ is large, I^* takes a small value indicating that only a small portion of the initiator was activated during the polymerization.

Table S12: O-ATRP performance for the organic photocatalysts.[13]

OPC	α (%)[a]	*Đ*[b]	I^{*c}	E_{ox}^0 (V vs SCE)	$E_{ox}^*(S_1)$ (V vs SCE)	$E_{ox}^*(T_1)$ (V vs SCE)	$\Phi(T_1)$[e]	τ_{DF} (ns)[f]
2Cz-BP	No 455 nm absorption			1.28	-1.82	-1.67	0.21 ± 0.15	3260 ± 360
2tCz-BP	70.0	1.61	0.36	1.05	-2.01	-1.88	0.17 ± 0.05	7900 ± 1300
4DP-IPN[d]	58.0	1.30	0.61	1.01	-1.58	-1.41	0.99 ± 0.13	75700 ± 8600
2PXZ-BP	3.0	1.62	0.02	0.79	-1.92	-1.73	(7.2 ± 2.2) × 10^{-4}	300 ± 20
2PTZ-BP	7.0	1.73	0.03	0.74	-2.47	-2.19	0.0011 ± 0.0003	310 ± 10

[a] Determined gravimetrically. [b] Determined by gel permeation chromatography equipped with refractive index detector using PMMA standards. [c] *I** is defined in the main text. [d] Carried out under the irradiation of two 3-W 515-nm LEDs. [e] Determined by TVAS measurement using OPC solutions in DMF in this work. [f] Determined via TCSPC measurements using OPC solutions in DMF in this work.

References

1. Roberts, G. M.; Marroux, H. J. B.; Grubb, M. P.; Ashfold, M. N. R.; Orr-Ewing, A. J., On the Participation of Photoinduced N–H Bond Fission in Aqueous Adenine at 266 and 220 nm: A Combined Ultrafast Transient Electronic and Vibrational Absorption Spectroscopy Study. *J. Phys. Chem. A* **2014,** *118* (47), 11211-11225.
2. Greetham, G. M.; Donaldson, P. M.; Nation, C.; Sazanovich, I. V.; Clark, I. P.; Shaw, D. J.; Parker, A. W.; Towrie, M., A 100 kHz Time-Resolved Multiple-Probe Femtosecond to Second Infrared Absorption Spectrometer. *Appl. Spectrosc.* **2016,** *70* (4), 645-653.
3. Greetham, G. M.; Sole, D.; Clark, I. P.; Parker, A. W.; Pollard, M. R.; Towrie, M., Time-resolved multiple probe spectroscopy. *Rev. Sci. Instrum.* **2012,** *83* (10), 103107.
4. Zhang, Q.; Huang, T.; He, X.; Zhao, Z.-R.; Zhao, X., Synthesis and properties of dicarbazolyltriphenylethylene-substituted fluorene derivatives exhibiting aggregation-induced emission enhancement. *Heterocycl. Commun.* **2017,** *23* (1), 15-21.
5. Qi, Y.; Wang, Y.; Yu, Y.; Liu, Z.; Zhang, Y.; Qi, Y.; Zhou, C., Exploring highly efficient light conversion agents for agricultural film based on aggregation induced emission effects. *J. Mater. Chme. C.* **2016,** *4* (47), 11291-11297.
6. Chen, C.; Liao, J.-Y.; Chi, Z.; Xu, B.; Zhang, X.; Kuang, D.-B.; Zhang, Y.; Liu, S.; Xu, J., Metal-free organic dyes derived from triphenylethylene for dye-sensitized solar cells: tuning of the performance by phenothiazine and carbazole. *J. Mater. Chem.* **2012,** *22* (18), 8994-9005.
7. Lee, S. Y.; Yasuda, T.; Yang, Y. S.; Zhang, Q.; Adachi, C., Luminous butterflies: efficient exciton harvesting by benzophenone derivatives for full-color delayed fluorescence OLEDs. *Angew. Chem. Int. Ed. Engl.* **2014,** *53* (25), 6402-6.
8. Kwon, Y.; Lee, J.; Noh, Y.; Kim, D.; Lee, Y.; Yu, C.; Roldao, J. C.; Feng, S.; Gierschner, J.; Wannemacher, R., et al., Formation and degradation of strongly reducing cyanoarene-based radical anions towards efficient radical anion-mediated photoredox catalysis. *Nat. Commun.* **2023,** *14* (1), 92.
9. Grubb, M. P.; Orr-Ewing, A. J.; Ashfold, M. N. R., KOALA: A program for the processing and decomposition of transient spectra. *Rev. Sci. Instrum.* **2014,** *85* (6), 064104.
10. Ishimatsu, R.; Matsunami, S.; Shizu, K.; Adachi, C.; Nakano, K.; Imato, T., Solvent effect on thermally activated delayed fluorescence by 1,2,3,5-tetrakis(carbazol-9-yl)-4,6-dicyanobenzene. *J. Phys. Chem. A* **2013,** *117* (27), 5607-12.
11. Sneha, M.; Thornton, G. L.; Lewis-Borrell, L.; Ryder, A. S. H.; Clark, I. P.; Cresswell, A. J.; Grayson, M. N.; Orr-Ewing, A. J., Photoredox-HAT Catalysis for Primary Amine α-C–H Alkylation: Mechanistic Insight with Transient Absorption Spectroscopy. *ACS Catal.* **2023,** *13* (12), 8004-8013.
12. Noda, H.; Chen, X.-K.; Nakanotani, H.; Hosokai, T.; Miyajima, M.; Notsuka, N.; Kashima, Y.; Brédas, J.-L.; Adachi, C., Critical role of intermediate electronic states for spin-flip processes in charge-transfer-type organic molecules with multiple donors and acceptors. *Nat. Mater.* **2019,** *18* (10), 1084-1090.
13. Singh, V. K.; Yu, C.; Badgujar, S.; Kim, Y.; Kwon, Y.; Kim, D.; Lee, J.; Akhter, T.; Thangavel, G.; Park, L. S., et al., Highly efficient organic photocatalysts discovered via a computer-aided-design strategy for visible-light-driven atom transfer radical polymerization. *Nature Catalysis* **2018,** *1* (10), 794-804.
